# A new optimization algorithm based on mimicking the voting process for leader selection

**DOI:** 10.7717/peerj-cs.976

**Published:** 2022-05-13

**Authors:** Pavel Trojovský, Mohammad Dehghani

**Affiliations:** 1 Department of Mathematics, Faculty of Science, University of Hradec Králové, Hrdaec Králové, Hradec Králové, Czech Republic

**Keywords:** Optimization, Optimization problem, Human-based metahurestic algorithm, Stochastic algorithms, Population-based algorithms, Applied mathematics, Voting process, Leader selection, Population matrix, Recurring process

## Abstract

Stochastic-based optimization algorithms are effective approaches to addressing optimization challenges. In this article, a new optimization algorithm called the Election-Based Optimization Algorithm (EBOA) was developed that mimics the voting process to select the leader. The fundamental inspiration of EBOA was the voting process, the selection of the leader, and the impact of the public awareness level on the selection of the leader. The EBOA population is guided by the search space under the guidance of the elected leader. EBOA’s process is mathematically modeled in two phases: exploration and exploitation. The efficiency of EBOA has been investigated in solving thirty-three objective functions of a variety of unimodal, high-dimensional multimodal, fixed-dimensional multimodal, and CEC 2019 types. The implementation results of the EBOA on the objective functions show its high exploration ability in global search, its exploitation ability in local search, as well as the ability to strike the proper balance between global search and local search, which has led to the effective efficiency of the proposed EBOA approach in optimizing and providing appropriate solutions. Our analysis shows that EBOA provides an appropriate balance between exploration and exploitation and, therefore, has better and more competitive performance than the ten other algorithms to which it was compared.

## Introduction

Optimization is an integral part of engineering, industry, technology, mathematics, and many other applications in science. Decision variables, constraints, and objective function are the three main parts of any optimization issue, where determining the values of decision variables while respecting the constraints to optimize the objective function is the main challenge of optimization (Ray & Liew, 2003).

Optimization problem solving approaches are included in two groups: deterministic and stochastic (Kozlov, Samsonov & Samsonova, 2016). Deterministic approaches, which include gradient-based and non-gradient-based techniques, have successful performance in handling convex and linear optimization problems. However, these approaches fail to meet real-world challenges with features such as convex behavior, nonlinear search space, high number of variables, complex objective function, high number of constraints, as well as NP-hard problems. Following the inability of deterministic approaches to address these types of issues, researchers have developed a new approach called stochastic optimization techniques. Metaheuristic algorithms are one of the most widely used stochastic approach techniques that are effective in optimization applications by using random operators, random scanning in the search space, and trial and error (Curtis & Robinson, 2019).

Simplicity of concept, ease of implementation, problem-independent, needlessness of the derivation process, and efficiency in complex problems are some of the advantages that have led to the popularity and applicability of metaheuristic algorithms (Gonzalez et al., 2022).

Metaheuristic algorithms have a nearly identical problem-solving process that begins with the production of a certain number of candidate solutions at random. Then, in a repetition-based process, the effect of the algorithm steps on these candidate solutions improves them. At the end of the implementation, the best-founded candidate solution is introduced as the solution to the problem (Toloueiashtian, Golsorkhtabaramiri & Rad, 2022).

It is important to note here that there is no guarantee that metaheuristic algorithms will be able to provide the best optimal solution known as the global optimal. This is due to the random nature of the search process of these algorithms. For this reason, the solution obtained from metaheuristic algorithms is called quasi-optimal (Yu, Semeraro & Matta, 2018).

The two indicators of exploration with the concept of global search and exploitation with the concept of local search are effective in the performance of metaheuristic algorithms in handling optimization problems and providing better quasi-optimal solutions (Mejahed & Elshrkawey, 2022). What has led researchers to develop numerous optimization methods is to achieve better solutions closer to the global optimal.

The main research question is whether there is a need to develop new metaheuristic algorithms given that countless algorithms have been developed so far. This question is answered with the concept of the No Free Lunch (NFL) theorem (Wolpert & Macready, 1997). The NFL theorem explains that the effective performance of an algorithm in solving a set of optimization problems does not create any presuppositions on the ability of that algorithm to provide similar performance in other optimization applications. In other words, it cannot be claimed that a particular metaheuristic algorithm performs best in the face of all optimization problems compared to all other optimization methods. The NFL theorem is the main incentive for the authors to design new optimization approaches that perform more effectively in solving optimization problems in a variety of applications. The NFL theorem has motivated the authors of this article to develop a new metaheuristic algorithm applicable in optimization challenges that is effective in providing solutions closer to the global optimization.

The novelty of this article is in introducing and designing a new metaheuristic algorithm named Election-Based Optimization Algorithm (EBOA), which its fundamental inspiration is the simulation of the voting process and the popular movement. The main contributions of this study are as follows:

• A novel human-based Election-Based Optimization Algorithm (EBOA) is proposed.
• The process of public movement and the electoral voting process are examined and then mathematically modeled in the EBOA design.
• The efficiency of EBOA in optimizing thirty-three objective functions (*i.e.,* unimodal, high-dimensional multimodal, and fixed-dimensional multimodal, and CEC 2019) is tested.
• The quality of EBOA results is compared with ten state-of-the-art metaheuristic algorithms.

The rest of the article is structured in such a way that the literature review is presented in ‘Lecture Review’. Then in ‘Election-Based Optimization Algorithm’ the proposed EBOA is introduced and modeled. Simulation studies are presented in ‘Results’. The discussion is provided in ‘Discussion’. Conclusions and several research directions for future studies are presented in ‘Conclusions’.

### Lecture review

Natural phenomena, the behavior of living things in nature, the biological sciences, genetic sciences, the laws of physics, the rules of the game, human behavior, and any evolutionary process that has an optimization process have been the source of inspiration in the design and development of metaheuristic algorithms. Accordingly, metaheuristic algorithms fall into nine groups: swarm-based, biology-based, physics-based, human-based, sport-based, math-based, chemistry-based, music-based, and the other hybrid approaches (Akyol & Alatas, 2017).

Behaviors of living organisms such as animals, birds, and insects have been the main source of ideas in the development of numerous swarm-based algorithms. The most common feature used in many swarm-based methods is the ability of living organisms to search for food sources. The most popular methods developed based on food search process modeling are the Particle Swarm Optimization (PSO) based on the search behavior of birds and fish (Kennedy & Eberhart, 1995), Ant Colony Optimization (ACO) based on ants search for the shortest path to food (Dorigo, Maniezzo & Colorni, 1996), Artificial Bee Colony (ABC) based on bee colony search behavior (Karaboga & Basturk, 2007), the Butterfly Optimization Algorithm (BOA) based on search and mating behavior of butterflies (Arora & Singh, 2019), and the Tunicate Search Algorithm (TSA) based on search behavior tonics (Kaur et al., 2020). The process of reproduction among bees and the scout bees search mechanism to find suitable new places for hives have been employed in the designing Fitness Dependent Optimizer (FDO) (Abdullah & Ahmed, 2019). Chimpanzee’s hunting strategy using operators such as emotional intelligence and sexual motivation has been the main source of inspiration in designing the Chimp Optimization Algorithm (ChOA) (Khishe & Mosavi, 2020).

Modeling of hunting strategies of living organisms in the wild has been a source of inspiration in designing various optimization approaches, including Grey Wolf Optimizer (GWO) based on gray wolf strategy (Mirjalili, Mirjalili & Lewis, 2014), Whale Optimization Algorithm (WOA) (Mirjalili & Lewis, 2016) based on humpback whales strategy, and Pelican Optimization Algorithm (POA) based on pelican behavior (Trojovský & Dehghani, 2022).

Applying the concepts of biology, genetics, and natural selection alongside random operators such as selection, crossover, and mutation has led to the development of biology-based algorithms. The process of reproduction, Darwin’s evolutionary theory, and natural selection are key concepts in the development of two widely used methods, the Genetic Algorithm (GA) (Goldberg & Holland, 1988) and the Differential Evolution (DE) algorithm (Storn & Price, 1997). The mechanism of the immune system in the face of diseases, viruses, and microbes has been the major inspiration in the development of the artificial immune system (AIS) method (Hofmeyr & Forrest, 2000).

Many phenomena, laws, and forces in physics science have been employed as inspiration sources for the development of physics-based metaheuristic algorithms. The phenomenon of melting and cooling of metals, which is known in physics as the refrigeration process, has been the main inspiration in the development of the Simulated Annealing (SA) approach (Van Laarhoven & Aarts, 1987). The phenomenon of the water cycle based on its physical changes in nature has inspired the Water Cycle Algorithm (WCA) (Eskandar et al., 2012). Gravitational force and Newton’s laws of motion have been the main concepts employed to introduce the method of Gravitational Search Algorithm (GSA) (Rashedi, Nezamabadi-Pour & Saryazdi, 2009). The application of Hook’s law and spring tensile force has been the main inspiration in Spring Search Algorithm (SSA) design (Dehghani et al., 2020a). Various physical theories and concepts have been the source of inspiration in the development of physics-based methods such as Multiverse Optimizer (MVO), inspired from cosmology concepts (Mirjalili, Mirjalili & Hatamlou, 2016), Big Bang-Big Crunch (BB-BC) inspired from Big Bang and Big Crunch theories (Erol & Eksin, 2006), Big Crunch Algorithm (BCA) inspired from Closed Universe theory (Kaveh & Talatahari, 2009), Integrated Radiation Algorithm (IRA) inspired from gravitational radiation concept in Einstein’s theory of general relativity (Chuang & Jiang, 2007), and Momentum Search Algorithm (MSA) inspired from momentum concept (Dehghani & Samet, 2020).

Behavior, thought, interactions, and collaborations in humans have been designed ideas in the development of human-based approaches. The most widely used human-based method is the Teaching-Learning-Based Optimization (TLBO) algorithm, which mimics the classroom learning environment and the interactions between students and teachers (Rao, Savsani & Vakharia, 2011). The competition between political parties and the efforts of parties to seize control of parliament is the source of inspiration in designing the Parliamentary Optimization Algorithm (POA) (Borji & Hamidi, 2009). The economic activities of the rich and the poor to gain wealth in society have been the main inspiration for the Poor and Rich Optimization (PRO) approach (Moosavi & Bardsiri, 2019). Influencing people in the community from the best successful people in the community has been the main idea of the Following Optimization Algorithm (FOA) (Dehghani, Mardaneh & Malik, 2020). The mechanism of admission of high school graduates to the university and the process of improving the educational level of students has been the main idea in designing the Learner Performance-based Behavior (LPB) algorithm (Rahman & Rashid, 2021). The cooperation of the members of a team to improve the performance of the team in performing their tasks and achieving the goal has been the main inspiration of the Teamwork Optimization Algorithm (TOA) (Dehghani & Trojovský, 2021). The efforts of human society to achieve felicity by changing and improving the thinking of individuals has been employed in the design of the Human Felicity Algorithm (HFA) (Veysari, 2022). The strategic movement of army troops during the war, using attack, defense, and troop relocation operations, has been a central idea in the design of War Strategy Optimization (WSO) (Ayyarao et al., 2022).

The rules governing various games, both individual and group, along with the activities of players, referees, coaches, and influential individuals, have been the main source of inspiration in the development of sport-based methods. The effort of the players in the tug-of-war competition have been the main idea in designing the Tug of War Optimization (TWO) technique (Kaveh & Zolghadr, 2016). The use of volleyball club interactions and the coaching process has been instrumental in designing the Volleyball Premier League (VPL) approach (Kaveh & Zolghadr, 2016). The players’ effort to find a hidden object was the main idea used in Hide Object Game Optimization (HOGO) (Dehghani et al., 2020b). The strategy that players and individuals use to solve the puzzle and arrange the puzzle pieces to complete it has been the source of inspiration in designing the Puzzle Optimization Algorithm (POA) (Zeidabadi & Dehghani, 2022).

Matheuristics (Boschetti et al., 2009) and the Base Optimization Algorithm (BOA) (Salem, 2012) are among math-based methods. Chemical Reaction Optimization (CRO) (Lam & Li, 2009) and the Artificial Chemical Reaction Optimization Algorithm (ACROA) (Alatas, 2011) are among chemistry-based methods. The Harmony Search Algorithm (HSA) (Geem, Kim & Loganathan, 2001) is music-based method. In addition, by combining metaheuristic algorithms with each other, researchers have developed hybrid metaheuristic approaches, including: the Sine-Cosine and Spotted Hyena-based Chimp Optimization Algorithm (*SSC*) (Dhiman, 2021) and the Hybrid Aquila Optimizer with Arithmetic Optimization Algorithm (AO-AOA) (Mahajan et al., 2022).

The literature review shows that numerous metaheuristic algorithms have been developed so far. However, according to the best knowledge of the literature, the voting process to determine the leader of the community has not yet been used in the design of any algorithm. This research gap motivated the authors of this article to develop a new human-based metaheuristic algorithm based on mathematical modeling of the electoral process and public movement.

### Election-based optimization algorithm

This section is dedicated to introducing the proposed Election-Based Optimization Algorithm (EBOA) and then mathematical modeling of it.

### Inspiration

An election is a process by which individuals in a community select a person from among the candidates. The person elected as the leader influences the situation of all members of that society, even those who did not vote for him. The more aware the community members are, the better they will be able to choose and vote for the better candidate. These expressed concepts of the election and voting process are employed in the design of the EBOA.

### Algorithm initialization

EBOA is a population-based metaheuristic algorithm whose members are community individuals. In the EBOA, each member of the population represents a proposed solution to the problem. From a mathematical point of view, the EBOA population is represented by a matrix called the population matrix using Eq. (1).

(1) \begin{eqnarray*}X={ \left[ \begin{array}{@{}c@{}} \displaystyle {X}_{1}\\ \displaystyle \vdots \\ \displaystyle {X}_{i}\\ \displaystyle \vdots \\ \displaystyle {X}_{N} \end{array} \right] }_{N\times m}={ \left[ \begin{array}{@{}ccccc@{}} \displaystyle {x}_{1,1}&\displaystyle \cdots &\displaystyle {x}_{1,j}&\displaystyle \cdots &\displaystyle {x}_{1,m}\\ \displaystyle \vdots &\displaystyle \ddots &\displaystyle \vdots &\displaystyle \iddots &\displaystyle \vdots \\ \displaystyle {x}_{i,1}&\displaystyle \cdots &\displaystyle {x}_{i,j}&\displaystyle \cdots &\displaystyle {x}_{i,m}\\ \displaystyle \vdots &\displaystyle \iddots &\displaystyle \vdots &\displaystyle \ddots &\displaystyle \vdots \\ \displaystyle {x}_{N,1}&\displaystyle \cdots &\displaystyle {x}_{N,j}&\displaystyle \cdots &\displaystyle {x}_{N,m} \end{array} \right] }_{N\times m},\end{eqnarray*}

where *X* refers to the EBOA population matrix, *X*_*i*_ refers to the *i*th EBOA member (*i.e.,* the proposed solution), *x*_*i*,*j*_ refers to the value of the *j*th problem variable specified by the *i*th EBOA member, *N* refers to EBOA population size, and *m* refers to number of decision variables.

The initial position of individuals in the search space is determined randomly according to Eq. (2).

(2) \begin{eqnarray*}{x}_{i,j}=l{b}_{j}+r\cdot \left( u{b}_{j}-l{b}_{j} \right) , i=1,2,\ldots ,N,j=1,2,\ldots ,m,\end{eqnarray*}

where *lb*_*j*_ and *ub*_*j*_ refer to the lower bound and upper bound of the *j*th variable, respectively, and *r* is a random number in the interval $ \left[ 0,1 \right] $.

Based on the values proposed by each EBO member for the problem variables, a value can be evaluated for the objective function. These evaluated values for the objective function of the problem are specified using a vector according to Eq. (3).

(3) \begin{eqnarray*}OF={ \left[ \begin{array}{@{}c@{}} \displaystyle O{F}_{1}\\ \displaystyle \vdots \\ \displaystyle O{F}_{i}\\ \displaystyle \vdots \\ \displaystyle O{F}_{N} \end{array} \right] }_{N\times 1}={ \left[ \begin{array}{@{}c@{}} \displaystyle OF({X}_{1})\\ \displaystyle \vdots \\ \displaystyle OF({X}_{i})\\ \displaystyle \vdots \\ \displaystyle OF({X}_{N}) \end{array} \right] }_{N\times 1},\end{eqnarray*}

where *OF* refers to the vector of obtained objective function values of EBOA population and *OF*_*i*_ refers to the obtained objective function value for the *i*th EBOA member. The values of the objective function are the criterion for measuring the quality of the proposed solutions in such a way that the best value of the objective function specifies the best member while the worst value of the objective function specifies the worst member.

### Mathematical model of EBOA

The main difference between metaheuristic algorithms is how members of the population are updated and the process that improves the proposed solutions in each iteration. The process of updating the algorithm population in EBOA has two phases of exploration and exploitation, which are discussed below.

Phase 1: Voting process and holding elections (exploration).

EBOA members, based on their awareness, participate in the election and vote for one of the candidates. People’s awareness can be considered as dependent on the quality and goodness of the value of the objective function. Accordingly, the awareness of individuals in the community is simulated using Eq. (4). In this awareness simulation process, individuals with better values of the objective function are more aware.

(4) \begin{eqnarray*}{A}_{i}= \left\{ \begin{array}{@{}ll@{}} \displaystyle \frac{O{F}_{i}-O{F}_{\mathrm{worst}}}{O{F}_{\mathrm{best}}-O{F}_{\mathrm{worst}}} , &\displaystyle O{F}_{\mathrm{best}}\not = O{F}_{\mathrm{worst}};\\ \displaystyle 1, &\displaystyle \mathrm{else}, \end{array} \right. \end{eqnarray*}

where *A*_*i*_ is the awareness of the *i*th EBOA member, *OF*_best_ and *OF*_worst_ are the best and worst values of the objective function, respectively. It should be noted that in minimization problems, *OF*_best_ is related to the minimum value of the objective function and *OF*_worst_ is related to the maximum value of the objective function, while in maximization problems, *OF*_best_ is related to the maximum value of the objective function and *OF*_worst_ is related to the minimum value of the objective function.

Among the members of the society, 10% of the most awareness individuals in the society are considered as election candidates. In the EBOA, it is assumed that the minimum number of candidates (*N*_*C*_) is equal to 2 (*i.e., N*_*C*_ ≥ 2), meaning that at least two candidates will register for the election.

The implementation of the voting process in EBOA is such that the level of awareness of each person is compared to a random number, if the level of awareness of a person is higher than that random number, the person is able to vote for the best candidate (known as *C*_1_). Otherwise, that person randomly votes for one of the other candidates. This voting process is mathematically modeled in Eq. (5).

(5) \begin{eqnarray*}{V}_{i}= \left\{ \begin{array}{@{}ll@{}} \displaystyle {C}_{1}, &\displaystyle {A}_{i}\gt r;\\ \displaystyle {C}_{k}, &\displaystyle \mathrm{else}, \end{array} \right. \end{eqnarray*}

where *V*_*i*_ refers to the vote of the *i*th person in the community, *C*_1_ refers to the best candidate, and *C*_*k*_ refers to the *k* th candidate, where *k* isa randomly selected number from the set $ \left\{ 2,3,\ldots ,{N}_{C} \right\} $.

At the end of the voting process, based on the counting of votes, the candidate who has received the highest number of votes is selected as elected (leader). This elected leader affects the situation of all members of the society and even those who did not vote for him. The position of individuals in the EBOA is updated under the influence and guidance of the elected leader. This leader directs the algorithm population to different areas in the search space and increases the EBOA’s exploration ability in the global search. The process of updating the EBOA population is led by the leader in such a way that firstly a new position is generated for each member. The newly generated position is acceptable for updating if it improves the value of the objective function. Otherwise, the corresponding member remains in the previous position. This update process in the EBOA is modeled using Eqs. (6) and (7).

(6) \begin{eqnarray*}{x}_{i,j}^{\mathrm{new},P1}& = \left\{ \begin{array}{@{}ll@{}} \displaystyle {x}_{i,j}+r\cdot ({L}_{j}-I\cdot {x}_{i,j}), &\displaystyle O{F}_{L}\lt O{F}_{i};\\ \displaystyle {x}_{i,j}+r\cdot ({x}_{i,j}-{L}_{j}), &\displaystyle \mathrm{else}, \end{array} \right. \end{eqnarray*}

(7) \begin{eqnarray*}{X}_{i}& = \left\{ \begin{array}{@{}ll@{}} \displaystyle {X}_{i}^{\mathrm{new}.P1}, &\displaystyle O{F}_{i}^{\mathrm{new},P1}\lt O{F}_{i};\\ \displaystyle {X}_{i}, &\displaystyle \mathrm{else}, \end{array} \right. \end{eqnarray*}

where ${X}_{i}^{\mathrm{new}.P1}$ refers to a new generated position for the *i*th EBOA member, ${x}_{i,j}^{\mathrm{new},P1}$ is its *j*th dimension, $O{F}_{i}^{\mathrm{new},P1}$ is its value of the objective function, *I* is an integer selected randomly from the values 1 or 2, *L* refers to the elected leader, *L*_*j*_ is its *j*th dimension, and *OF*_*L*_ is its objective function value.

Phase 2: Public movement to raise awareness (exploitation).

The awareness of the people of the society has a great impact on their correct decisions in the election and voting process. In addition to the leader’s influence on people’s awareness, every person’s thoughts and activities can increase that person’s awareness. From a mathematical point of view, a better solution may be identified based on a local search adjacent to any proposed solution. Thus, the activities of community members to increase their awareness, lead to an increase in the EBOA’s exploitation ability in the local search and find better solutions to the problem. To simulate this local search process, a random position is considered in the neighborhood of each member in the search space. The objective function of the problem is then evaluated based on this new situation to determine if this new situation is better than the existing situation of that member. If the new position has a better value for the objective function, the local search is successful and the position of the corresponding member is updated. Improving the value of the objective function will increase that person’s awareness for better decision-making in the next election (in the next iteration). This update process to increase people’s awareness in the EBOA is modeled using Eqs. (8) and (9).

(8) \begin{eqnarray*}{x}_{i,j}^{\mathrm{new},P2}& ={x}_{i,j}+ \left( 1-2r \right) \cdot R\cdot \left( 1- \frac{t}{T} \right) \cdot {x}_{i,j},\end{eqnarray*}

(9) \begin{eqnarray*}{X}_{i}& = \left\{ \begin{array}{@{}ll@{}} \displaystyle {X}_{i}^{\mathrm{new},P2}, &\displaystyle O{F}_{i}^{\mathrm{new},P2}\lt O{F}_{i};\\ \displaystyle {X}_{i}, &\displaystyle \mathrm{else}, \end{array} \right. \end{eqnarray*}

where ${X}_{i}^{\mathrm{new}.P2}$ refers to a new generated position for the *i*th EBOA member, ${x}_{i,j}^{\mathrm{new},P2}$ is its *j*th dimension, $O{F}_{i}^{new,P2}$ is its value of the objective function, *R* is the constant equals to 0.02, *t* refers to iteration contour, and *T* refers to maximum number of iterations.

### Repetition process, pseudocode, and flowchart of EBOA

An EBOA iteration is completed after updating the status of all members of the population. The EBOA enters the next iteration with the newly updated values, and the population update process is repeated based on the first and second phases according to Eqs. (4) to (9) until the last iteration. Upon completion of the full implementation of the algorithm, EBOA introduces the best proposed solution found during the algorithm iterations as the solution to the problem. The EBOA steps are summarized as follows:

Start.

Step 1: Specify the given optimization problem information: objective function, constraints, and a number of decision variables.

Step 2: Adjust the number of iterations of the algorithm (*T*) and the population size (*N*).

Step 3: Initialize the EBOA population at random and evaluate the objective function.

Step 4: Update the best and worst members of the EBOA population.

Step 5: Calculate the awareness vector of the community.

Step 6: Determine the candidates from the EBOA population.

Step 7: Hold the voting process.

Step 8: Determine the elected leader based on the vote count.

Step 9: Update the position of EBOA members based on elected leader guidance in the search space.

Step 10: Update the position of EBOA members based on the concept of local search and public movement to raise awareness.

Step 11: Save the best EBOA member as the best candidate solution so far.

Step 12: If the iterations of the algorithm are over, go to the next step, otherwise go back to Step 4.

Step 13: Print the best-obtained candidate solution in the output.

End.

**Figure 1:**
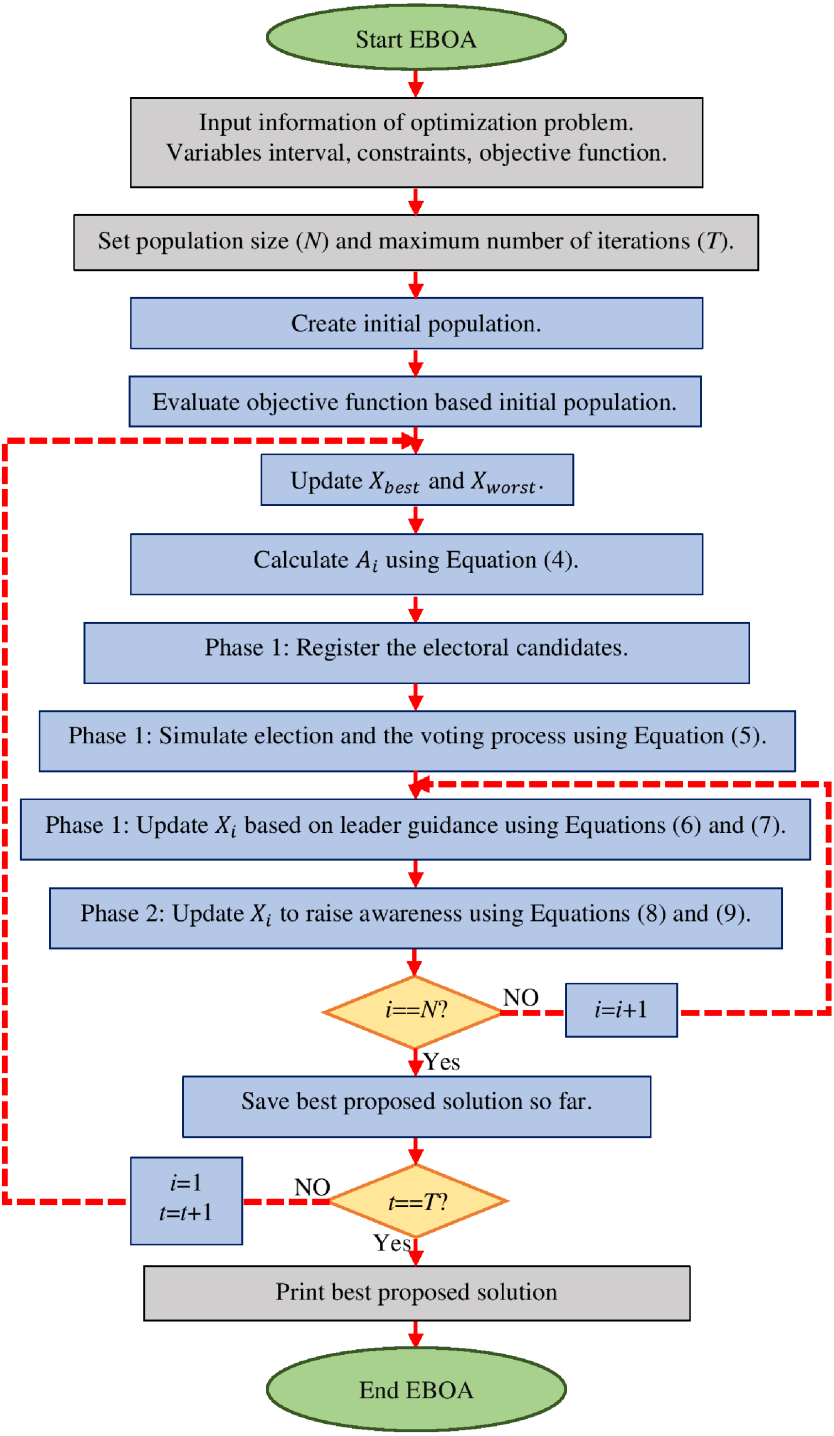
Flowchart of EBOA.

The flowchart of all steps of implementation of the EBOA is specified in Fig. 1 and its pseudocode is presented in Algorithm 1.

**Table:** Pseudocode of EBOA. Algorithm 1.

Start EBOA.
Input problem information: variables, objective function, and constraints.
Set EBOA population size (*N*) and iterations (*T*).
Generate the initial population matrix at random.
Evaluate the objective function.
For *t*= 1 to *T*
Update best and worst population members.
Phase 1: Voting process and holding elections (exploration).
Calculate *A* using Eq. (4).
Determine candidates based on awareness criteria.
Simulate holding election and voting using Eq. (5).
Count the votes and determine the election winner as leader.
For *i*= 1 to *N*
Calculate ${X}_{i}^{\mathrm{new},{P}_{1}}$ using Eq. (6).
Update *X*_*i*_ using Eq. (7).
Phase 2: Public movement to raise awareness (exploitation).
Calculate ${X}_{i}^{\mathrm{new},{P}_{2}}$ using Eq. (8).
Update *X*_*i*_ using Eq. (9).
end
Save best proposed solution so far.
end
Output best quasi-optimal solution obtained with the EBOA.
End EBOA.

### Computational complexity of EBOA

This subsection is devoted to examining the computational complexity of the EBOA. The computational complexity of EBOA initialization, including random population generation and initial evaluation of the objective function, is equal to *O*(*Nm*)where *N* is the size of the EBOA population and *m* is the number of problem variables. Holding the election and updating the EBOA population in the first phase has the computational complexity equal to *O*(*NmT*) where *T* is the number of iterations. Population update based on the second phase of EBOA to increase people’s awareness is equal to *O*(*NmT*). Accordingly, the total computational complexity of EBOA is equal to *O*(*Nm*(1 + 2*T*)).

## Results

This section is dedicated to analyzing EBOA performance in optimization and its ability to provide solutions to problems. Thirty-three objective functions of different types have been selected to evaluate different aspects of the proposed approach. Information and details of these benchmark functions are specified in Tables 1, 2, 3 and 4. The reasons for selecting these objective functions are as follows: functions F1 to F7 are selected as a unimodal type. These types of functions have only one extremum in their search space and are suitable in this regard to evaluate the EBOA’s exploitation ability in local search and converge to this optimal position. Therefore, the reason for choosing unimodal functions is to evaluate the exploitation potential of EBOA. The high-dimensional multimodal functions F8 to F13 include numerous local extremums in their search space in addition to the main extremum. These local optimal situations may cause the algorithm to fail. This feature has adapted the functions F8 to F13 to analyze the EBOA’s exploration ability in global search and determine whether the proposed approach is able to bypass local optimal locations and identify the original optimal location. Therefore, the reason for choosing high-dimensional multimodal functions is to evaluate the EBOA exploration capability. Fixed-dimensional multimodal functions F14 to F23 have fewer local optimal locations in their search space. These types of functions are great criteria for simultaneously measuring exploration and exploitation in optimization methods. Therefore, the reason for choosing fixed-dimensional multimodal functions is to evaluate the EBOA’s ability to strike the balance between the powers of exploration and exploitation. In addition to the twenty-three classic F1 to F23 objective functions, EBOA performance on ten complexes CEC 2019 suite test functions (known as the 100-Digit Challenge Test Functions) is also tested. More information and details about CEC 2019 test functions are available in Price et al. (2018).

**Table 1:** Information of unimodal objective functions.

Objective function		Range	Dimensions	*F*_min_
1.	${F}_{1} \left( x \right) ={\mathop{\sum }\nolimits }_{i=1}^{m}{x}_{i}^{2}$	$ \left[ -100,100 \right] $	30	0
2.	${F}_{2} \left( x \right) ={\mathop{\sum }\nolimits }_{i=1}^{m} \left\vert {x}_{i} \right\vert +{\mathop{\prod }\nolimits }_{i=1}^{m} \left\vert {x}_{i} \right\vert $	$ \left[ -10,10 \right] $	30	0
3.	${F}_{3} \left( x \right) ={\mathop{\sum }\nolimits }_{i=1}^{m}{ \left( {\mathop{\sum }\nolimits }_{j=1}^{i}{x}_{i} \right) }^{2}$	$ \left[ -100,100 \right] $	30	0
4.	${F}_{4} \left( x \right) =\mathrm{max} \left\{ \left\vert {x}_{i} \right\vert ,1\leq i\leq m \right\} $	$ \left[ -100,100 \right] $	30	0
5.	${F}_{5} \left( x \right) ={\mathop{\sum }\nolimits }_{i=1}^{m-1} \left[ 100{ \left( {x}_{i+1}-{x}_{i}^{2} \right) }^{2}+{ \left( {x}_{i}-1 \right) }^{2} \right] $	$ \left[ -30,30 \right] $	30	0
6.	${F}_{6} \left( x \right) ={\mathop{\sum }\nolimits }_{i=1}^{m} \left( [{x}_{i}+0.5] \right) ^{2}$	$ \left[ -100,100 \right] $	30	0
7.	${F}_{7} \left( x \right) ={\mathop{\sum }\nolimits }_{i=1}^{m}i{x}_{i}^{4}+\mathrm{random}(0,1)$	$ \left[ -1.28,1.28 \right] $	30	0

**Table 2:** Information of high-dimensional multimodal objective functions.

Objective function		Range	Dimensions	*F*_min_
8.	${F}_{8} \left( x \right) ={\mathop{\sum }\nolimits }_{i=1}^{m}-{x}_{i}\sin (\sqrt{{|}{x}_{i}{|}})$	$ \left[ -500,500 \right] $	30	−1.2569E +04
9.	${F}_{9} \left( x \right) ={\mathop{\sum }\nolimits }_{i=1}^{m}[{x}_{i}^{2}-10\cos \left( 2\pi {x}_{i} \right) +10]$	$ \left[ -5.12,5.12 \right] $	30	0
10.	${F}_{10} \left( x \right) =-20\exp \left( -0.2\sqrt{ \frac{1}{m} {\mathop{\sum }\nolimits }_{i=1}^{m}{x}_{i}^{2}} \right) -\exp \left( \frac{1}{m} {\mathop{\sum }\nolimits }_{i=1}^{m}\mathit{cos} \left( 2\pi {x}_{i} \right) \right) +20+e$	$ \left[ -32,32 \right] $	30	0
11.	${F}_{11} \left( x \right) = \frac{1}{4000} {\mathop{\sum }\nolimits }_{i=1}^{m}{x}_{i}^{2}-{\mathop{\prod }\nolimits }_{i=1}^{m}cos \left( \frac{{x}_{i}}{\sqrt{i}} \right) +1$	$ \left[ -600,600 \right] $	30	0
12.	$\begin{array}{@{}l@{}} \displaystyle {F}_{12} \left( x \right) = \frac{\pi }{m} \left\{ 10\sin \left( \pi {y}_{1} \right) \right. \left. +{\mathop{\sum }\nolimits }_{i=1}^{m}{ \left( {y}_{i}-1 \right) }^{2} \left[ 1+10\sin 2 \left( \pi {y}_{i+1} \right) \right] +{ \left( {y}_{n}-1 \right) }^{2} \right\} +{\mathop{\sum }\nolimits }_{i=1}^{m}u \left( {x}_{i},10,100,4 \right) \\ \displaystyle u \left( {x}_{i},a,i,n \right) = \left\{ \begin{array}{@{}l@{}} \displaystyle k{ \left( {x}_{i}-a \right) }^{n},{x}_{i}\gt -a; \\ \displaystyle 0,-a\leq {x}_{i}\leq a; \\ \displaystyle k{ \left( -{x}_{i}-a \right) }^{n},{x}_{i}\lt -a. \end{array} \right. \end{array}$	$ \left[ -50,50 \right] $	30	0
13.	${F}_{13}(x)=0.1\{ \sin 2(3\pi {x}_{1})+{\mathop{\sum }\nolimits }_{i=1}^{m}({x}_{i}-1)^{2}[1+\sin 2(3\pi {x}_{i}+1)]+({x}_{n}-1)^{2}[1+\sin 2(2\pi {x}_{m})]\} +{\mathop{\sum }\nolimits }_{i=1}^{m}u({x}_{i},5,100,4)$	[ − 50, 50]	30	0

**Table 3:** Information of fixed-dimensional multimodal objective functions.

Objective function		Range	Dimensions	*F*_min_
14.	${F}_{14} \left( x \right) ={ \left( \frac{1}{500} +{\mathop{\sum }\nolimits }_{j=1}^{25} \frac{1}{j+{\mathop{\sum }\nolimits }_{i=1}^{2}{ \left( {x}_{i}-{a}_{ij} \right) }^{6}} \right) }^{-1}$	$ \left[ -65.53,65.53 \right] $	2	0.998
15.	${F}_{15} \left( x \right) ={\mathop{\sum }\nolimits }_{i=1}^{11}{ \left[ {a}_{i}- \frac{{x}_{1}({b}_{i}^{2}+{b}_{i}{x}_{2})}{{b}_{i}^{2}+{b}_{i}{x}_{3}+{x}_{4}} \right] }^{2}$	$ \left[ -5,5 \right] $	4	0.00030
16.	${F}_{16} \left( x \right) =4{x}_{1}^{2}-2.1{x}_{1}^{4}+ \frac{1}{3} {x}_{1}^{6}+{x}_{1}{x}_{2}-4{x}_{2}^{2}+4{x}_{2}^{4}$	$ \left[ -5,5 \right] $	2	−1.0316
17.	${F}_{17} \left( x \right) ={ \left( {x}_{2}- \frac{5.1}{4{\pi }^{2}} {x}_{1}^{2}+ \frac{5}{\pi } {x}_{1}-6 \right) }^{2}+10 \left( 1- \frac{1}{8\pi } \right) \cos {x}_{1}+10$	[ − 5, 10] × [0, 15]	2	0.398
18.	${F}_{18} \left( x \right) = \left[ 1+{ \left( {x}_{1}+{x}_{2}+1 \right) }^{2} \left( 19-14{x}_{1}+3{x}_{1}^{2}-14{x}_{2}+6{x}_{1}{x}_{2}+3{x}_{2}^{2} \right) \right] \times [30+{ \left( 2{x}_{1}-3{x}_{2} \right) }^{2}\times (18-32{x}_{1}+12{x}_{1}^{2}+48{x}_{2}-36{x}_{1}{x}_{2}+27{x}_{2}^{2})]$	$ \left[ -5,5 \right] $	2	3
19.	${F}_{19} \left( x \right) =-{\mathop{\sum }\nolimits }_{i=1}^{4}{c}_{i}\exp (-{\mathop{\sum }\nolimits }_{j=1}^{3}{a}_{ij}{ \left( {x}_{j}-{P}_{ij} \right) }^{2})$	$ \left[ 0,1 \right] $	3	−3.86
20.	${F}_{20} \left( x \right) =-{\mathop{\sum }\nolimits }_{i=1}^{4}{c}_{i}\exp (-{\mathop{\sum }\nolimits }_{j=1}^{6}{a}_{ij}{ \left( {x}_{j}-{P}_{ij} \right) }^{2})$	$ \left[ 0,1 \right] $	6	−3.22
21.	${F}_{21} \left( x \right) =-{\mathop{\sum }\nolimits }_{i=1}^{5}{ \left[ \left( X-{a}_{i} \right) { \left( X-{a}_{i} \right) }^{T}+6{c}_{i} \right] }^{-1}$	$ \left[ 0,10 \right] $	4	−10.1532
22.	${F}_{22} \left( x \right) =-{\mathop{\sum }\nolimits }_{i=1}^{7}{ \left[ \left( X-{a}_{i} \right) { \left( X-{a}_{i} \right) }^{T}+6{c}_{i} \right] }^{-1}$	$ \left[ 0,10 \right] $	4	−10.4029
23.	${F}_{23} \left( x \right) =-{\mathop{\sum }\nolimits }_{i=1}^{10}{ \left[ \left( X-{a}_{i} \right) { \left( X-{a}_{i} \right) }^{T}+6{c}_{i} \right] }^{-1}$	$ \left[ 0,10 \right] $	4	−10.5364

**Table 4:** Information on complex CEC 2019 objective functions.

Objective function		Range	Dimensions	*F*_min_
1.	Storn’s Chebyshev Polynomial Fitting Problem	$ \left[ -8192,8192 \right] $	9	1
2.	Inverse Hilbert Matrix Problem	$ \left[ -16384,16384 \right] $	16	1
3.	Lennard-Jones Minimum Energy Cluster	$ \left[ -4,4 \right] $	18	1
4.	Rastrigin’s Function	[ − 100, 100]	10	1
5.	Griewangk’s Function	[ − 100, 100]	10	1
6.	Weierstrass Function	[ − 100, 100]	10	1
7.	Modified Schwefel’s Function	[ − 100, 100]	10	1
8.	Expanded Schaffer’s F6 Function	[ − 100, 100]	10	1
9.	Happy Cat Function	[ − 100, 100]	10	1
10.	Ackley Function	[ − 100, 100]	10	1

The quality of the EBOA optimization results is compared with ten state-of-the-art metaheuristic algorithms including (i) the most widely used and oldest methods: GA, PSO, (ii) most cited methods from 2009 to 2014: GSA, TLBO, GWO, (iii) Recently published and widely used methods from 2016 to 2021: WOA, MPA, LPB, FDO, and TSA. As noted in the literature, numerous optimization methods have been developed to date. Comparing the proposed EBOA approach with all of these methods, while possible, generates a hug deal of data. Among the metaheuristic algorithms developed, some methods have attracted more attention due to their high efficiency. For this reason, in this study, the ten mentioned metaheuristic algorithms that have been most considered and used have been selected to compare with the performance of EBOA. The values set for the control parameters of these metaheuristics are listed in Table 5.

**Table 5:** Adjusted values for competitor metaheuristic algorithms.

Algorithm	Parameter	Value
GA		
	Type	Real coded.
	Selection	Roulette wheel (Proportionate).
	Crossover	Whole arithmetic (Probability = 0.8, $\alpha \in \left[ -0.5,1.5 \right] $)
	Mutation	Gaussian (Probability = 0.05).
PSO		
	Topology	Fully connected.
	Cognitive and social constant	$ \left( {C}_{1},{C}_{2} \right) = \left( 2,2 \right) $.
	Inertia weight	Linear reduction from 0.9 to 0.1
	Velocity limit	10% of dimension range.
GSA		
	Alpha, *G*_0_, *R*_norm_, *R*_power_	20, 100, 2, 1
TLBO		
	*T*_*F*_: teaching factor	${T}_{F}=\text{round} \left[ (1+rand) \right] $,
	Random number	*rand* is a random number in interval $ \left[ 0,1 \right] .$
GWO		
	Convergence parameter (*a*)	*a*: Linear reduction from 2 to 0.
WOA		
	Convergence parameter (*a*)	*a*: Linear reduction from 2 to 0.
	*r*	*r* is a vector of random numbers in $ \left[ 0,1 \right] .$
	*l*	*l* is a random number in $ \left[ -1,1 \right] .$
TSA		
	P_min_ and P _max_	1 and 4
	*c*_1_, *c*_2_, *c*_3_	Random numbers lie in the range from 0 to 1.
MPA		
	Constant number	*P* = 0.5,
	Random vector	*R* is a vector of uniform random numbers in the interval $ \left[ 0,1 \right] .$
	Fish Aggregating Devices (*FADs*)	*FADs* = 0.2
	Binary vector	*U* = 0 or 1
FDO		
	Weight factor *wf*	*wf* is either 0 or 1
	*r*	*r* is a vector of random numbers in 0,1.
LPB		
	Crossover Percentage	*pc* = 0.6,
	Number of Offsprings (Parnets)	*nc* = 2 ⋅*round*(*pc*⋅*nPop*/2),
	Mutation Percentage	*pm* = 0.3,
	Number of Mutants	*nm* = *round*(*pm*⋅*nPop*),
	Mutation Rate	*mu* = 0.03,
	Divide probability	*dp* = 0.5,
		*beta* = 8
		*gamma* = 0.05

The EBOA and ten competitor metaheuristics are each employed in twenty independent implementations to solve the objective functions F1 to F23, while each implementation contains 1,000 repetitions. The termination condition can be based on various criteria such as number of iterations, number of function evaluations, the error between several consecutive iterations, and other cases. In this study, the termination condition is considered based on the number of iterations. The experiments are performed in the Matlab R2020a version in the environment of Microsoft Windows 10 with 64 bits on the Core i-7 processor with 2.40 GHz and 6 GB memory. Simulation results and performance of metaheuristic algorithms are reported using five indicators: mean, best proposed solution, standard deviation (std), median, and rank.

### Evaluation unimodal objective function

The results of applying EBOA and ten competitor metaheuristic algorithms to optimize F1 to F7 unimodal functions are reported in Table 6. The optimization outputs show the EBOA has provided the global optimal in solving the F1, F3, and F6 functions. EBOA is the first best optimizer in solving F2, F4, F5, and F7. The simulation results show that in handling the F1 to F7 functions, the EBOA performed better than the ten competitor metaheuristic algorithms and ranked first.

**Table 6:** Optimization results of EBOA and competitor metaheuristics on the unimodal function.

		**EBOA**	**FDO**	**LPB**	**MPA**	**TSA**	**WOA**	**GWO**	**TLBO**	**GSA**	**PSO**	**GA**
F_1_	Mean	0	1.27E−31	0.6059539	1.71E−18	8.21E−33	1.59E−09	1.09E−58	1.34E−59	2.03E−17	1.77E−05	13.24063
	Best	0	3.29E−34	0.1656726	5.92E−26	1.14E−62	1.09E−16	7.73E−61	9.36E−61	8.20E−18	2.00E−10	5.59388
	std	0	1.96E−31	0.2883118	6.76E−18	2.53E−32	3.22E−09	4.09E−58	2.05E−59	7.10E−18	5.86E−05	5.72729
	Median	0	2.61E−32	0.552771	1.63E−19	6.83E−40	1.09E−09	1.08E−59	4.69E−60	1.78E−17	9.93E−07	11.04546
	Rank	1	5	10	6	4	8	3	2	7	9	11
F_2_	Mean	1.30E−261	3.26E−16	0.2088937	2.78E−09	5.02E−39	0.538136	1.30E−34	5.55E−35	2.37E−08	0.341139	2.479432
	Best	8.30E−271	3.19E−17	0.1002184	4.25E−18	8.26E−43	0.461308	1.55E−35	1.32E−35	1.59E−08	0.001741	1.591248
	std	0	3.87E−16	0.0757105	1.08E−08	1.72E−38	0.048065	2.20E−34	4.71E−35	3.96E−09	0.669594	0.642843
	Median	3.50E−265	1.79E−16	0.1831153	3.18E−11	8.26E−41	0.545056	6.38E−35	4.37E−35	2.33E−08	0.130114	2.463873
	Rank	1	5	8	6	2	10	4	3	7	9	11
F_3_	Mean	0	0.8016065	5116.4258	0.377013	3.20E−19	9.94E−08	7.41E−15	7.01E−15	279.3468	589.4942	1536.915
	Best	0	0.0148424	2992.3972	0.032038	7.29E−30	1.74E−12	4.75E−20	1.21E−16	81.91242	1.614937	1014.689
	std	0	1.8545893	1727.1652	0.201758	9.90E−19	3.87E−07	1.90E−14	1.27E−14	112.3057	1524.007	367.2108
	Median	0	0.2027959	5275.2084	0.378658	9.81E−21	1.74E−08	1.59E−16	1.86E−15	291.441	54.15445	1510.715
	Rank	1	7	11	6	2	5	4	3	8	9	10
F_4_	Mean	5.30E−260	0.8309008	2.9453299	3.66E−08	2.01E−22	5.10E−05	1.26E−14	1.58E−15	3.25E−09	3.963461	2.094271
	Best	3.10E−266	0.211467	2.0418994	3.42E−17	1.87E−52	7.34E−06	3.43E−16	6.42E−16	2.09E−09	1.604522	1.389849
	std	0	0.5440558	0.5030838	6.45E−08	5.96E−22	5.74E−05	2.32E−14	7.14E−16	7.50E−10	2.204083	0.337011
	Med	2.10E−262	0.7476137	2.8820789	3.03E−08	3.13E−27	3.45E−05	7.30E−15	1.54E−15	3.34E−09	3.260791	2.09854
	Rank	1	8	10	6	2	7	4	3	5	11	9
F_5_	Mean	25.91771	45.546797	163.70642	42.49778	28.76746	41.15923	26.86099	145.6667	36.10723	50.26311	310.4313
	Best	24.94581	19.180105	95.107228	41.58682	28.53831	39.3088	25.21377	120.7932	25.83811	3.647051	160.5013
	std	0.433265	37.836937	41.043291	0.615521	0.364773	0.489502	0.884088	19.73905	32.46252	36.52379	120.4473
	Median	26.13112	22.222915	166.09256	42.49068	28.54102	41.3088	26.70967	142.8987	26.07475	28.69395	279.5174
	Rank	1	7	10	6	3	5	2	9	4	8	11
F_6_	Mean	0	4.93E−21	0.05	0.390872	3.84E−20	2.53E−09	0.642334	0.45	0	20.2502	14.5501
	Best	0	1.92E−23	0	0.274582	6.74E−26	1.95E−15	1.57E−05	0	0	5	6.00042
	std	0	9.71E−21	0.2236068	0.080282	1.50E−19	4.05E−09	0.301088	0.510418	0	12.77311	5.835177
	Median	0	1.41E−21	0	0.406648	6.74E−21	1.95E−09	0.621487	0	0	19	13.5
	Rank	1	2	5	6	3	4	8	7	1	10	9
F_7_	Mean	4.77E−05	0.8072237	0.0718038	0.002182	0.000276	0.01946	0.000819	0.00313	0.020692	0.113415	0.00568
	Best	9.87E−07	0.2679768	0.0318638	0.001429	0.000104	0.002027	0.000248	0.001362	0.01006	0.029593	0.002111
	std	4.40E−05	0.3625883	0.0221458	0.000466	0.000123	0.004115	0.000503	0.001351	0.01136	0.045868	0.002433
	Median	3.56E−05	0.8273515	0.0719665	0.00218	0.000367	0.020272	0.000629	0.002912	0.016996	0.107872	0.005365
	Rank	1	11	9	4	2	7	3	5	8	10	6
Sum rank		7	45	63	40	18	46	28	32	40	66	67
Mean rank		1	6.4285	9	5.7142	2.5714	6.5714	4	4.5714	5.7142	9.4285	9.5714
Total rank		1	6	8	5	2	7	3	4	5	9	10

### Evaluation of high-dimensional multimodal objective functions

The optimization results of F8 to F13 functions obtained from the implementation of EBOA and ten competing metaheuristic algorithms are released in Table 6. EBOA is able to converge to the global optimum in handling F9 and F11 functions. In optimizing the F10, F12, F13, and F14 functions, what is evident from the simulation results is that EBOA is the first best optimizer in these functions. In optimizing the F8 function, after GA and TLBO, the proposed EBOA is the third best optimizer of this function. What can be deduced from the results of Table 7 is that EBOA has a higher capability in optimizing high-dimensional multimodal functions compared to ten competitor algorithms and is ranked first as the best optimizer in functions 8 to F13.

**Table 7:** Optimization results of EBOA and competitor metaheuristics on the high-dimensional multimodal function.

		**EPOA**	**FDO**	**LPB**	**MPA**	**TSA**	**WOA**	**GWO**	**TLBO**	**GSA**	**PSO**	**GA**
F_8_	Mean	−7149.45	−6742.4711	−11057.297	−3652.09	−5669.56	−1633.55	−5885.02	−7803.47	−2849.03	−6908.54	−8184.3
	Best	−8600.95	−7688.9971	−11972.938	−4419.9	−5706.3	−2358.57	−7227.05	−9103.77	−3969.23	−8500.59	−9717.68
	std	720.2391	385.42421	340.85937	474.608	21.84579	374.5924	984.4547	986.5806	540.379	836.6452	795.1826
	Median	−7123.95	−6794.0493	−11028.692	−3632.65	−5669.63	−1649.72	−5774.63	−7735.22	−2671.33	−7098.95	−8117.25
	Rank	4	6	1	9	8	11	7	3	10	5	2
F_9_	Mean	0	12.106148	0.4418522	152.6934	0.005888	3.666025	8.53E−15	10.67763	16.26778	57.06189	62.41204
	Best	0	5.7972799	0.0558848	128.2306	0.004776	1.78099	0	9.873963	4.974795	27.85883	36.86623
	std	0	4.3697476	0.2628465	15.18316	0.000696	1.07177	2.08E−14	0.397117	4.658816	16.51737	15.21563
	Median	0	10.939576	0.3549152	154.6214	0.005871	3.78099	0	10.88657	15.42242	55.22468	61.67858
	Rank	1	7	4	11	3	5	2	6	8	9	10
F_10_	Mean	1.24E−15	8.76E−12	0.2378952	8.31E−10	6.38E−11	0.279162	1.71E−14	0.263208	3.57E−09	2.154699	3.221863
	Best	8.88E−16	1.22E−13	0.1155122	1.68E−18	8.14E−15	0.013128	1.51E−14	0.156316	2.64E−09	1.155151	2.757203
	std	1.09E−15	2.47E−11	0.0933506	2.80E−09	2.60E−10	0.146961	3.15E−15	0.072865	5.27E−10	0.549446	0.361797
	Median	8.88E−16	2.53E−12	0.2136625	1.05E−11	1.10E−13	0.312835	1.51E−14	0.261541	3.64E−09	2.170083	3.120322
	Rank	1	3	7	5	4	9	2	8	6	10	11
F_11_	Mean	0	0.0167647	0.5041482	0	1.55E−06	0.105702	0.003753	0.587689	3.737598	0.046293	1.230221
	Best	0	0	0.2403442	0	4.23E−15	0.08107	0	0.310117	1.519288	7.29E−09	1.140551
	std	0	0.0174995	0.19818	0	3.38E−06	0.007345	0.007344	0.169117	1.670282	0.051834	0.062756
	Median	0	0.0091183	0.4827497	0	8.77E−07	0.10701	0	0.582026	3.424268	0.029473	1.227231
	Rank	1	4	7	1	2	6	3	8	10	5	9
F_12_	Mean	2.71E−07	0.027568	0.0050244	0.082559	0.050164	1.557746	0.037211	0.020551	0.036283	0.480672	0.047027
	Best	1.63E−07	9.02E−23	0.0003721	0.077912	0.035428	0.56726	0.019295	0.002031	5.57E−20	0.000145	0.018364
	std	5.25E−08	0.0463417	0.009498	0.002386	0.009855	0.4596	0.013875	0.028645	0.060866	0.602582	0.028483
	Median	2.70E−07	3.21E−07	0.0010168	0.082111	0.050935	1.56726	0.032991	0.015181	1.48E−19	0.1556	0.04179
	Rank	1	4	2	9	8	11	6	3	5	10	7
F_13_	Mean	3.88E−06	5.49E−05	0.0307545	0.565254	2.658778	0.338392	0.576327	0.329124	0.002085	0.508412	1.208556
	Best	2.00E−06	4.55E−21	0.0064812	0.280295	2.63175	0.332688	0.297822	0.038266	1.18E−18	9.99E−07	0.49809
	std	9.01E−07	0.000202	0.0215577	0.187817	0.009796	0.001343	0.170359	0.198939	0.005476	1.251681	0.333754
	Median	3.79E−06	2.09E−17	0.0276489	0.579874	2.66175	0.338688	0.578323	0.282784	2.14E−18	0.043997	1.218096
	Rank	1	2	4	8	11	6	9	5	3	7	10
Sum rank		9	26	25	43	36	48	29	33	42	46	49
Mean rank		1.5	4.3333333	4.1666667	7.1666667	6	8	4.8333333	5.5	7	7.6666667	8.1666667
Total rank		1	3	2	8	6	10	4	5	7	9	11

**Table 8:** Optimization results of EBOA and competitor metaheuristics on the fixed-dimensional multimodal functions.

		**EBOA**	**FDO**	**LPB**	**MPA**	**TSA**	**WOA**	**GWO**	**TLBO**	**GSA**	**PSO**	**GA**
F_14_	Mean	0.998004	1.3459133	0.998004	0.998998	1.798757	1.043798	3.740858	2.264292	3.591435	2.173601	0.99867
	Best	0.998004	0.9980038	0.998004	0.998137	0.998004	0.998004	0.998004	0.998391	0.999508	0.998004	0.998004
	std	9.23E−14	0.4864376	1.10E−10	0.000324	0.527414	0.204528	3.969726	1.149621	2.778791	2.936536	0.002471
	Median	0.998004	0.9980038	0.998004	0.999138	1.912608	0.998004	2.982105	2.275231	2.986658	0.998004	0.998027
	Rank	1	5	1	3	6	4	10	8	9	7	2
F_15_	Mean	0.000308	0.0003138	0.0085214	0.003936	0.000408	0.003719	0.00637	0.003169	0.002402	0.001684	0.005395
	Best	0.000307	0.0003075	0.0004878	0.00027	0.000264	0.000441	0.000307	0.002206	0.000805	0.000307	0.000775
	std	3.30E−07	2.61E−05	0.0092568	0.005051	7.59E−05	0.001248	0.009401	0.000394	0.001195	0.004932	0.0081
	Median	0.000308	0.0003075	0.0022942	0.0027	0.00039	0.00441	0.000308	0.003185	0.002311	0.000307	0.002074
	Rank	1	2	11	8	3	7	10	6	5	4	9
F_16_	Mean	−1.03163	−1.0316285	−1.0316273	−1.03157	−1.03158	−1.03158	−1.03161	−1.03161	−1.03161	−1.03161	−1.0316
	Best	−1.03163	−1.0316285	−1.0316285	−1.0316	−1.03161	−1.0316	−1.03163	−1.03163	−1.03163	−1.03163	−1.03163
	std	1.14E−10	7.75E−10	2.19E−06	4.42E−05	4.09E−05	3.78E−05	3.78E−05	3.78E−05	3.78E−05	3.78E−05	4.92E−05
	Median	−1.03163	−1.0316285	−1.0316281	−1.0316	−1.0316	−1.0316	−1.03163	−1.03163	−1.03163	−1.03163	−1.03162
	Rank	1	2	3	7	6	6	4	4	4	4	5
F_17_	Mean	0.397887	0.3978875	0.3979073	0.399302	0.400093	0.405055	0.397894	0.397892	0.397892	0.785448	0.436972
	Best	0.397887	0.3978874	0.3978875	0.39757	0.398052	0.399405	0.397887	0.397887	0.397887	0.397887	0.397888
	std	3.28E−09	3.04E−07	2.95E−05	0.003672	0.00448	0.003664	1.02E−05	1.02E−05	1.02E−05	0.721752	0.140745
	Median	0.397887	0.3978874	0.3978944	0.39782	0.399052	0.40466	0.397888	0.397887	0.397887	0.397915	0.397925
	Rank	1	2	5	6	7	8	4	3	3	10	9
F_18_	Mean	3	3	3.0000232	3.000032	3.093107	3.000228	3.000042	3.000031	3.000031	3.000031	4.359425
	Best	3	3	3.0000003	3	2.999974	3.000149	3	3	3	3	3.000001
	std	0	1.49E−11	2.64E−05	7.69E−05	0.031851	0.000126	7.76E−05	7.69E−05	7.69E−05	7.69E−05	6.035694
	Median	3	3	3.0000109	3	3.103419	3.000149	3.000007	3	3	3	3.001083
	Rank	1	1	2	4	7	6	5	3	3	3	8
F_19_	Mean	−3.86278	−3.86278	−3.86278	−3.86264	−3.80654	−3.8616	−3.86211	−3.86132	−3.86272	−3.86272	−3.85428
	Best	−3.86278	−3.86278	−3.86278	−3.8627	−3.8366	−3.86276	−3.86278	−3.8625	−3.86278	−3.86278	−3.86278
	std	3.38E−07	5.74E−06	2.05E−07	0.000142	0.015257	0.003062	0.001704	0.001374	0.000142	0.000142	0.014852
	Median	−3.86278	−3.86278	−3.86278	−3.8627	−3.8066	−3.86266	−3.86275	−3.86187	−3.86278	−3.86278	−3.86226
	Rank	1	1	1	3	8	5	4	6	2	2	7
F_20_	Mean	−3.322	−3.3219846	−3.274437	−3.32105	−3.31947	−3.23224	−3.25234	−3.20112	−3.32195	−3.2619	−2.82386
	Best	−3.322	−3.321995	−3.3219951	−3.3213	−3.3212	−3.31342	−3.32199	−3.26174	−3.322	−3.322	−3.31342
	std	9.99E−08	1.16E−05	0.0597588	0.000147	0.003069	0.035652	0.076565	0.031823	0.000122	0.070623	0.385958
	Median	−3.322	−3.3219895	−3.3219932	−3.3211	−3.32058	−3.2424	−3.26231	−3.20744	−3.322	−3.32166	−2.96828
	Rank	1	2	6	4	5	9	8	10	3	7	11
F_21_	Mean	−10.1532	−10.150391	−5.642384	−9.95429	−5.40202	−7.40498	−9.64509	−9.19003	−5.14855	−5.38916	−4.30394
	Best	−10.1532	−10.153199	−10.153196	−10.1532	−7.50209	−7.48159	−10.1532	−9.66387	−10.1532	−10.1532	−7.82781
	std	2.33E−06	0.003942	3.4984609	0.532557	0.967922	0.03346	1.561937	0.120744	3.054458	3.019762	1.740798
	Median	−10.1532	−10.152212	−3.8690264	−10.1532	−5.50209	−7.40159	−10.1526	−9.1532	−3.64784	−5.10077	−4.16197
	Rank	1	2	7	3	8	6	4	5	10	9	11
F_22_	Mean	−10.4029	−10.134399	−7.01164	−10.2858	−5.9134	−8.6996	−10.4024	−10.0485	−10.0846	−7.63218	−5.11734
	Best	−10.4029	−10.402939	−10.402941	−10.4029	−9.06249	−10.4029	−10.4028	−10.4029	−10.4029	−10.4029	−9.11064
	std	2.18E−06	1.1783516	3.5358695	0.245334	1.754912	1.356173	0.000474	0.398327	1.423122	3.541608	1.969599
	Median	−10.4029	−10.401725	−7.7657081	−10.4027	−5.06249	−8.81649	−10.4025	−10.1836	−10.4029	−10.4019	−5.0294
	Rank	1	4	9	3	10	7	2	6	5	8	11
F_23_	Mean	−10.5364	−10.532661	−6.4463091	−10.1407	−9.80971	−10.0215	−10.1301	−9.26415	−10.5363	−6.16472	−6.56203
	Best	−10.5364	−10.536408	−10.536409	−10.5364	−10.3683	−10.5364	−10.5363	−10.534	−10.5364	−10.5364	−10.2216
	std	3.63E−06	0.0062871	3.8555684	1.140111	1.606403	0.355828	1.814366	1.676549	0.000386	3.734897	2.617187
	Median	−10.5364	−10.535362	−4.5055122	−10.5364	−10.3613	−10.0003	−10.5359	−9.67172	−10.5364	−4.50535	−6.5629
	Rank	1	3	10	4	7	6	5	8	2	11	9
Sum rank		10	24	55	45	67	64	56	59	46	65	82
Mean rank		1	2.4	5.5	4.5	6.7	6.4	5.6	5.9	4.6	6.5	8.2
Total rank		1	2	5	3	10	8	6	7	4	9	11

### Evaluation of fixed-dimensional multimodal objective functions

The results obtained from the implementation of EBOA and ten competitor metaheuristic algorithms on F14 to F23 functions are presented in Table 8. What emerges from the simulation output is that EBOA is the first best optimizer to handle F14 to F23 functions. Analysis and comparison of the obtained results indicate that the proposed EBOA approach has a superior performance over ten metaheuristic algorithms and among them, it has the first rank of the best optimizer.

The performance of the EBOA and the ten competitor metaheuristic algorithms implemented on the F1 to F23 objective functions are shown in Fig. 2 as the boxplot. For visual analysis of the ability to achieve the searched solution, Figs. 3 to 11 show the convergence curves of the EBOA and ten other competing algorithms in optimizing a number of objective functions.

**Figure 2:**
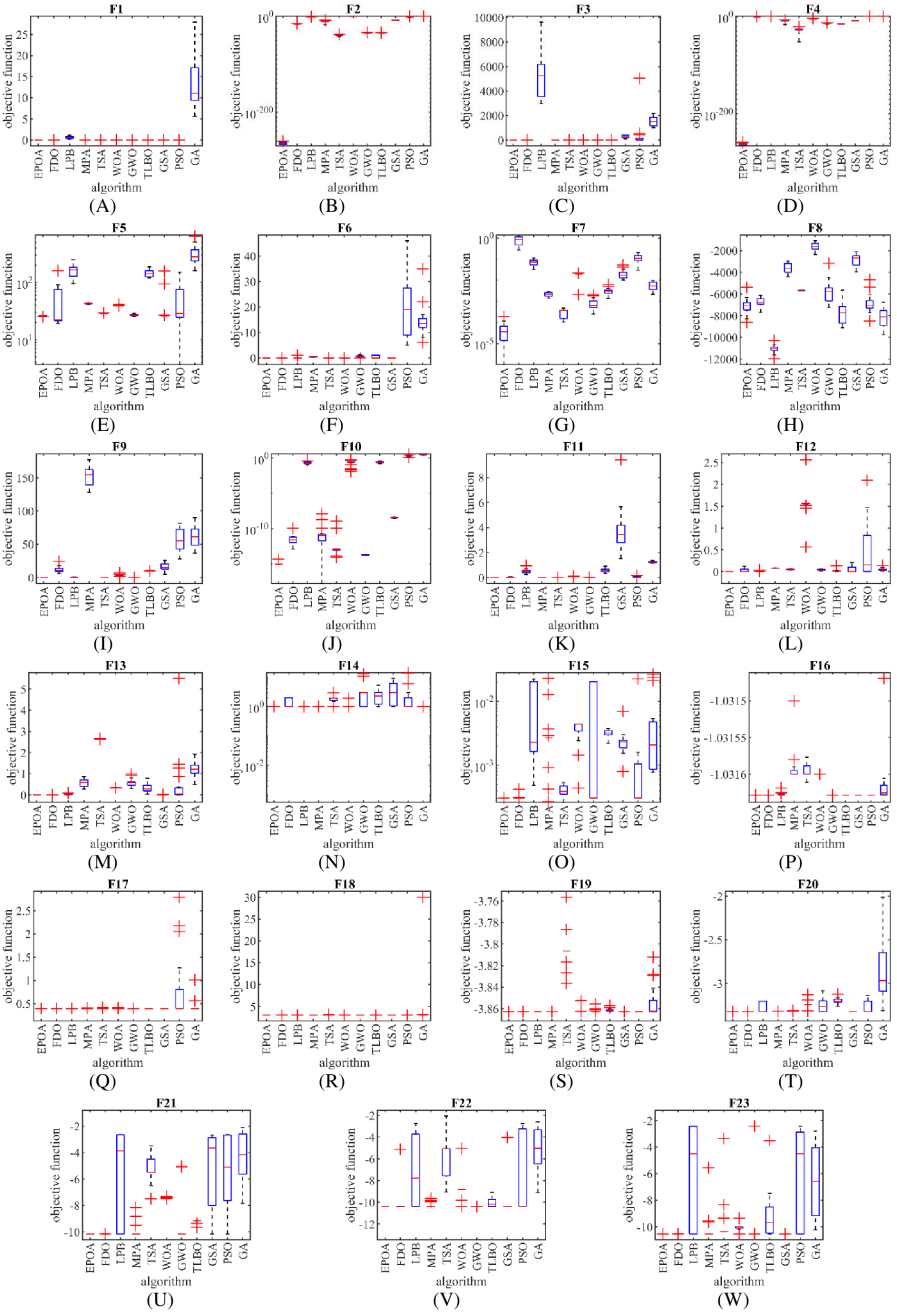
(A–W) Boxplot diagram of EPOA and ten metaheuristic algorithms performances on F1 to F23.

**Figure 3:**
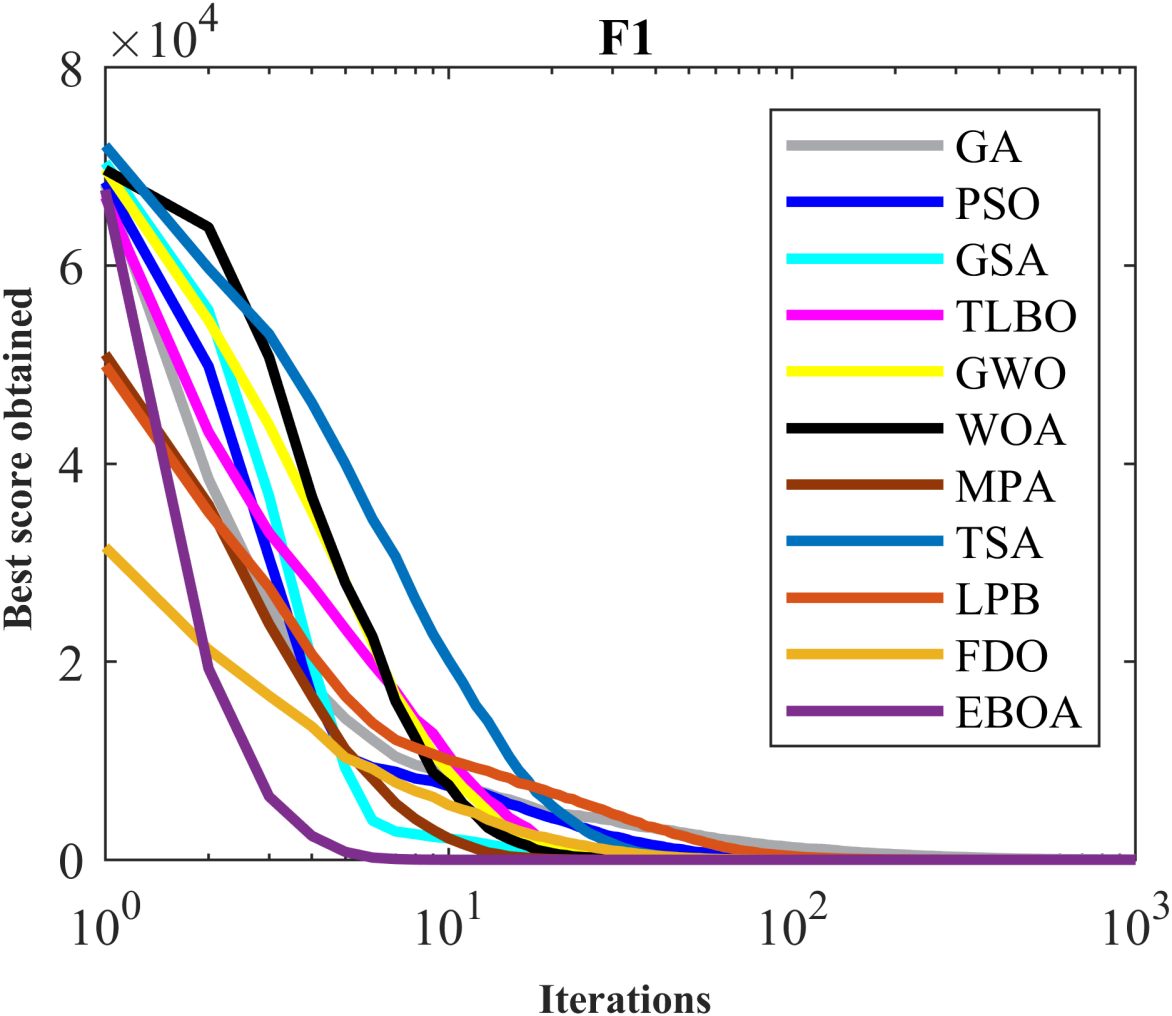
Convergence curves of EPOA and competitor algorithms on F1.

**Figure 4:**
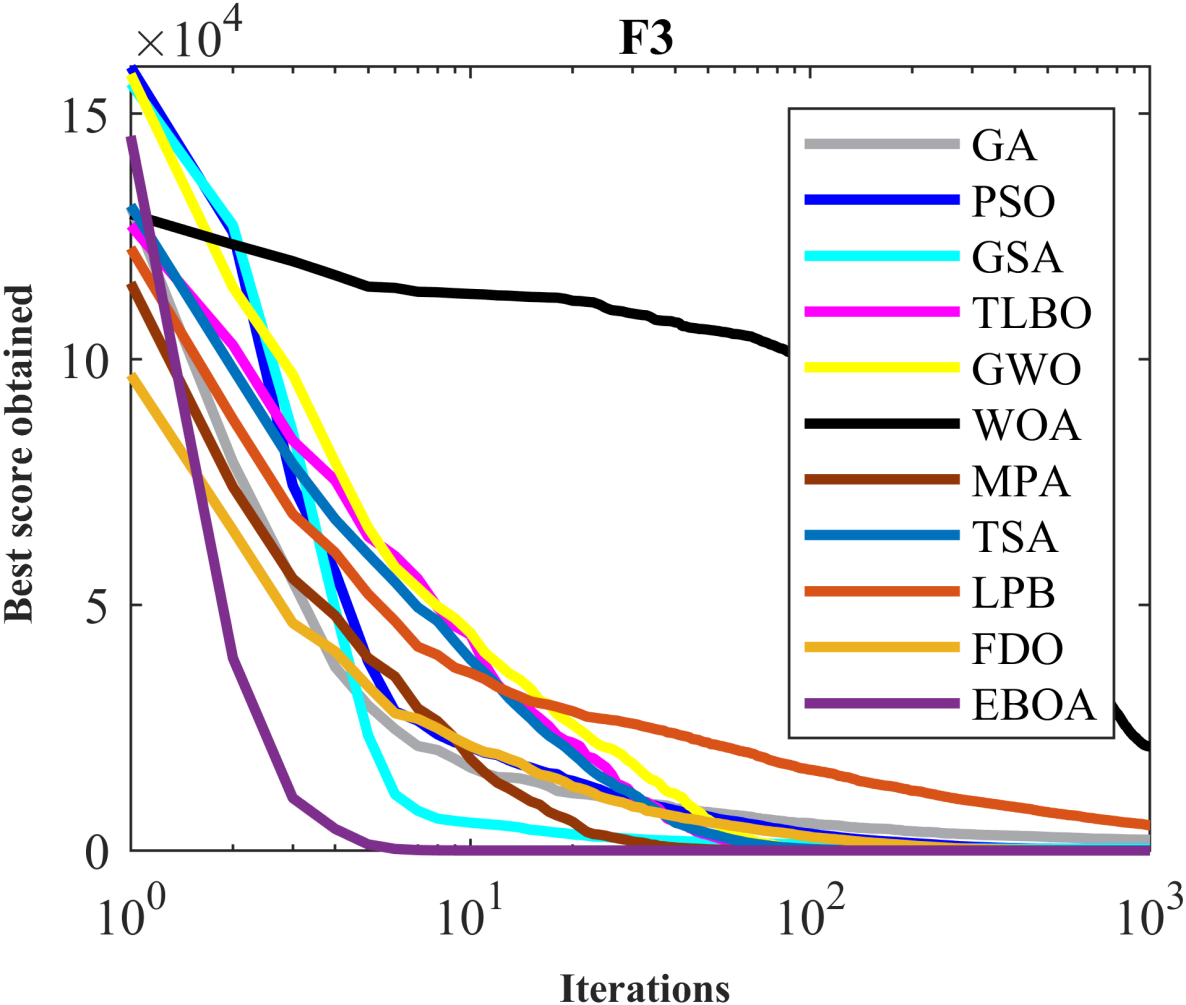
Convergence curves of EPOA and competitor algorithms on F3.

**Figure 5:**
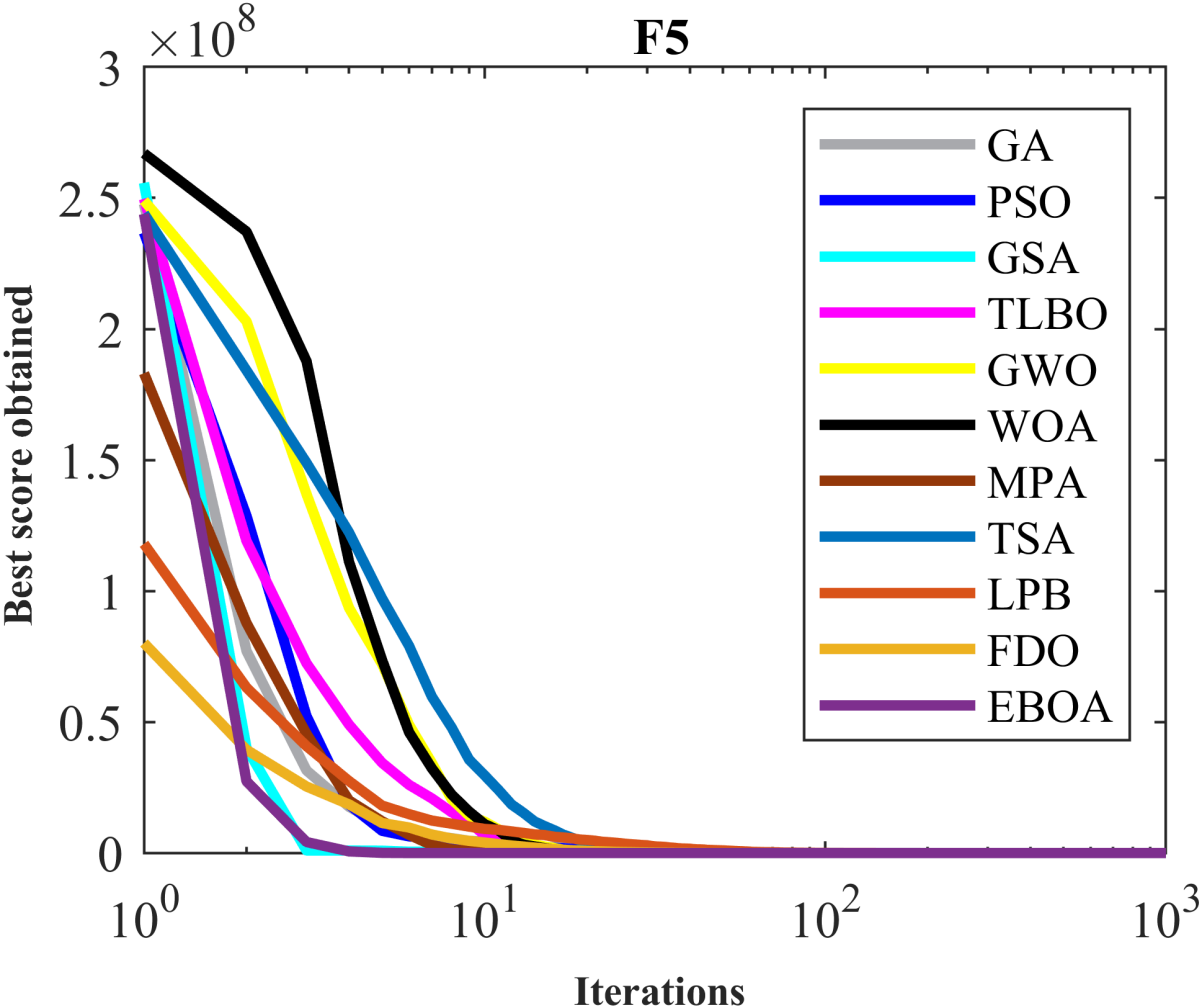
Convergence curves of EPOA and competitor algorithms on F5.

**Figure 6:**
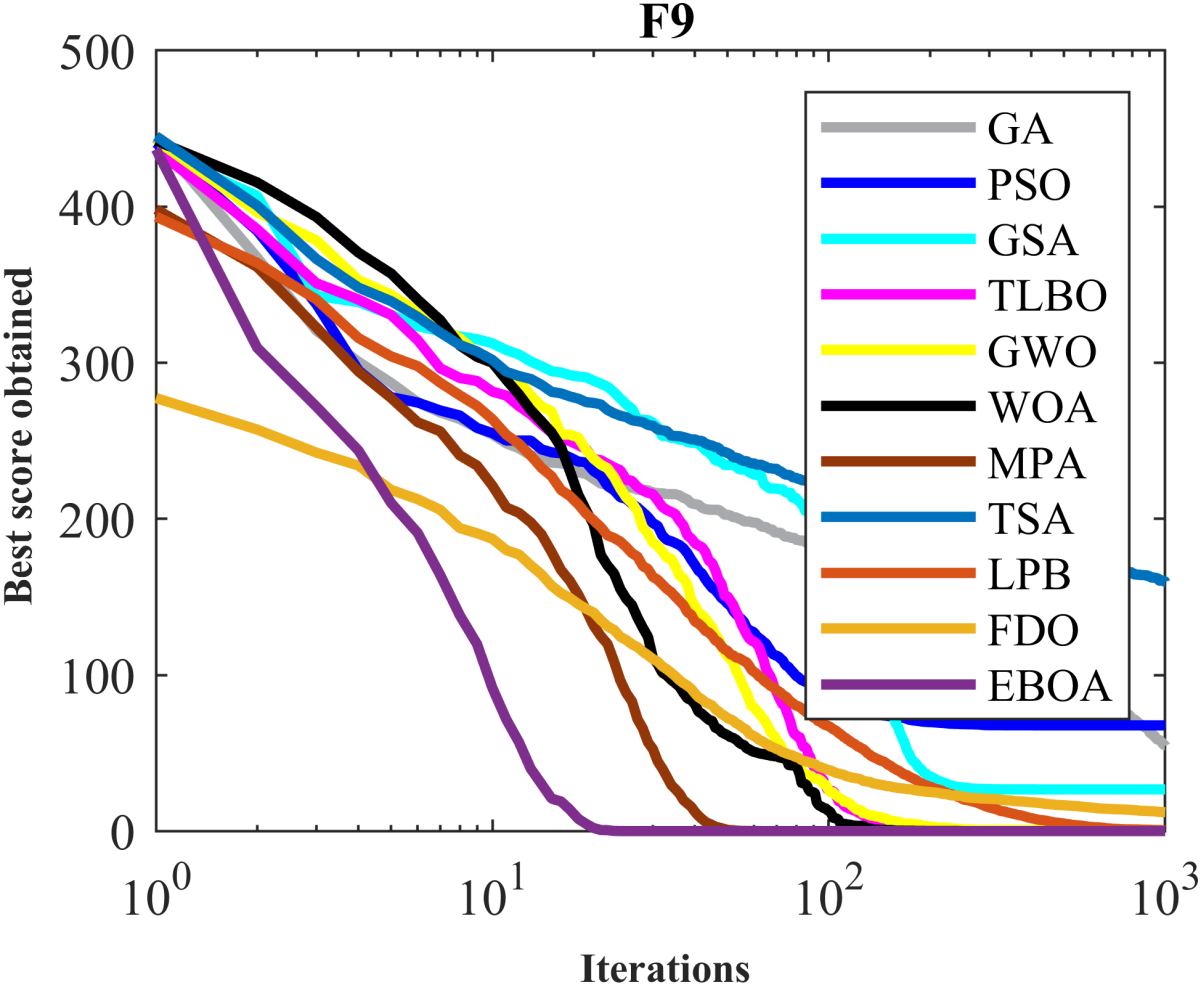
Convergence curves of EPOA and competitor algorithms on F9.

**Figure 7:**
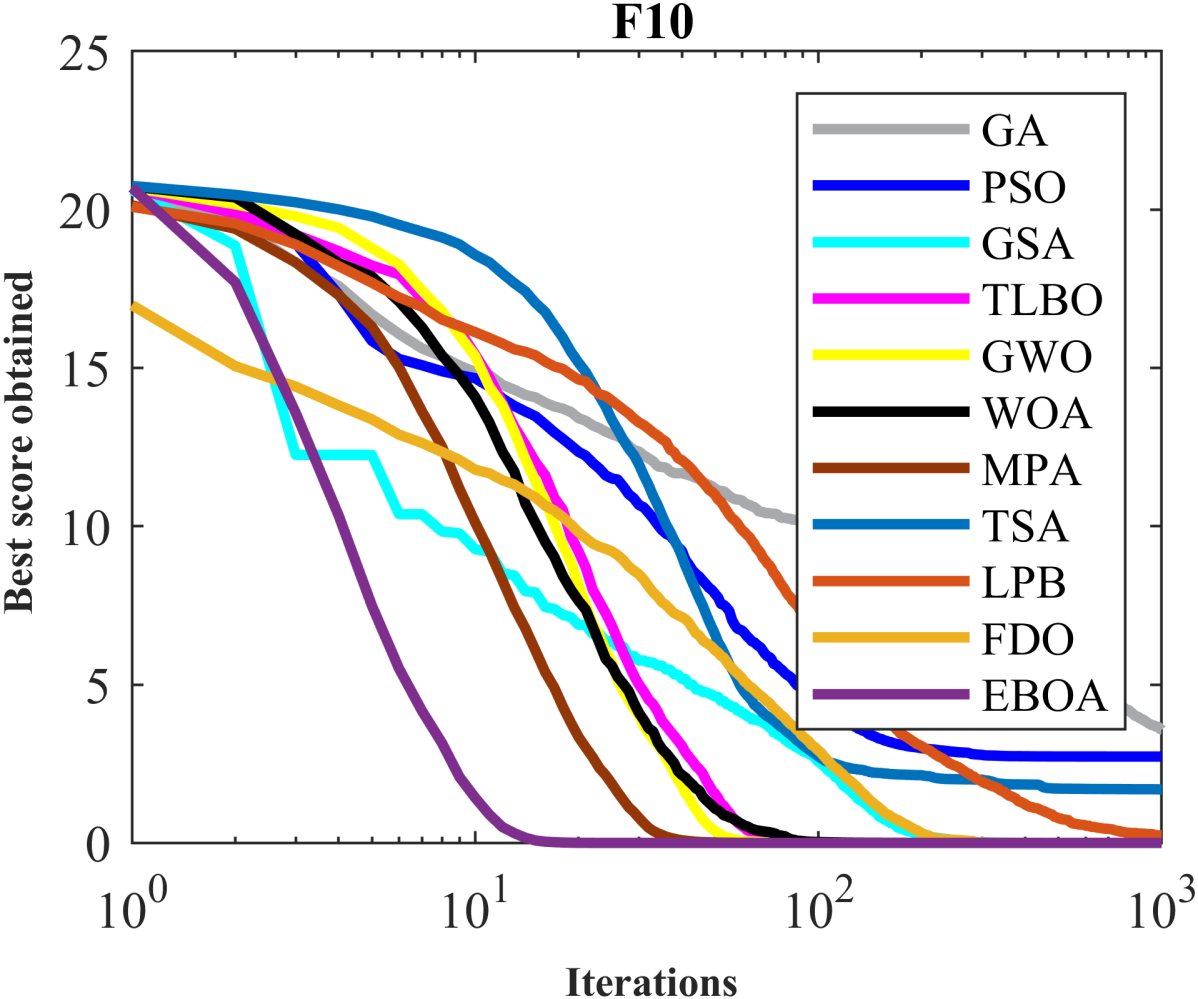
Convergence curves of EPOA and competitor algorithms on F10.

**Figure 8:**
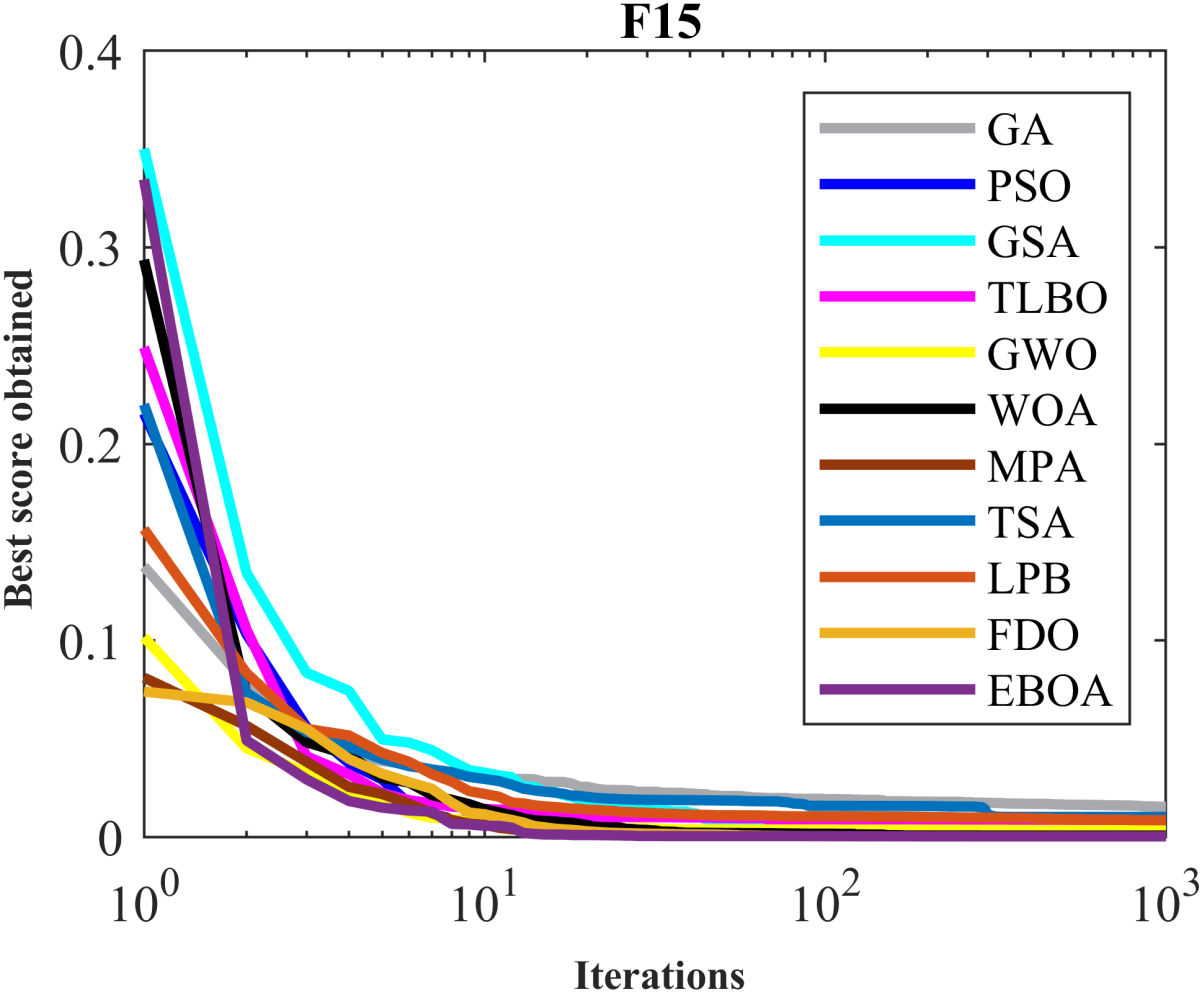
Convergence curves of EPOA and competitor algorithms on F15.

**Figure 9:**
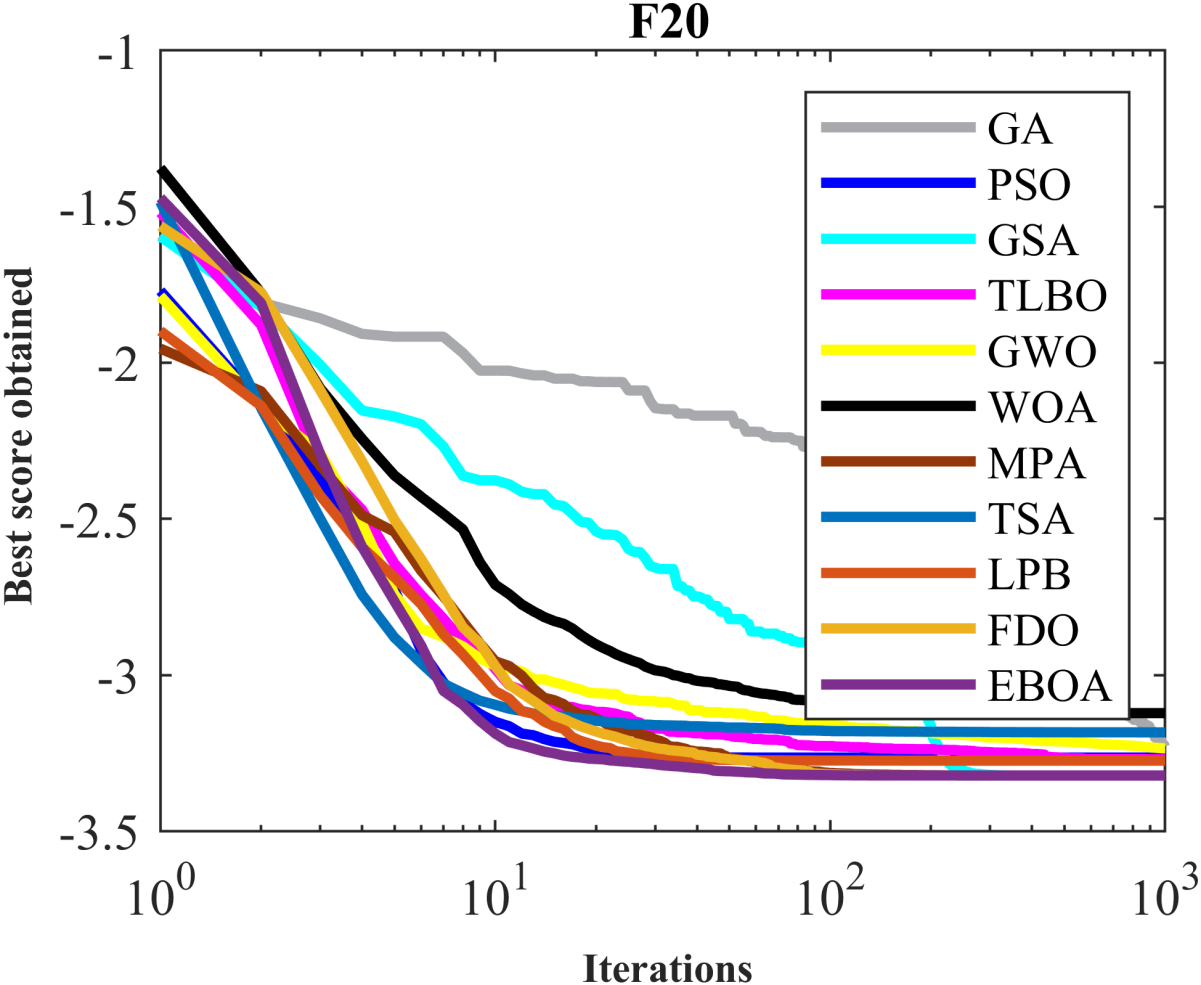
Convergence curves of EPOA and competitor algorithms on F20.

**Figure 10:**
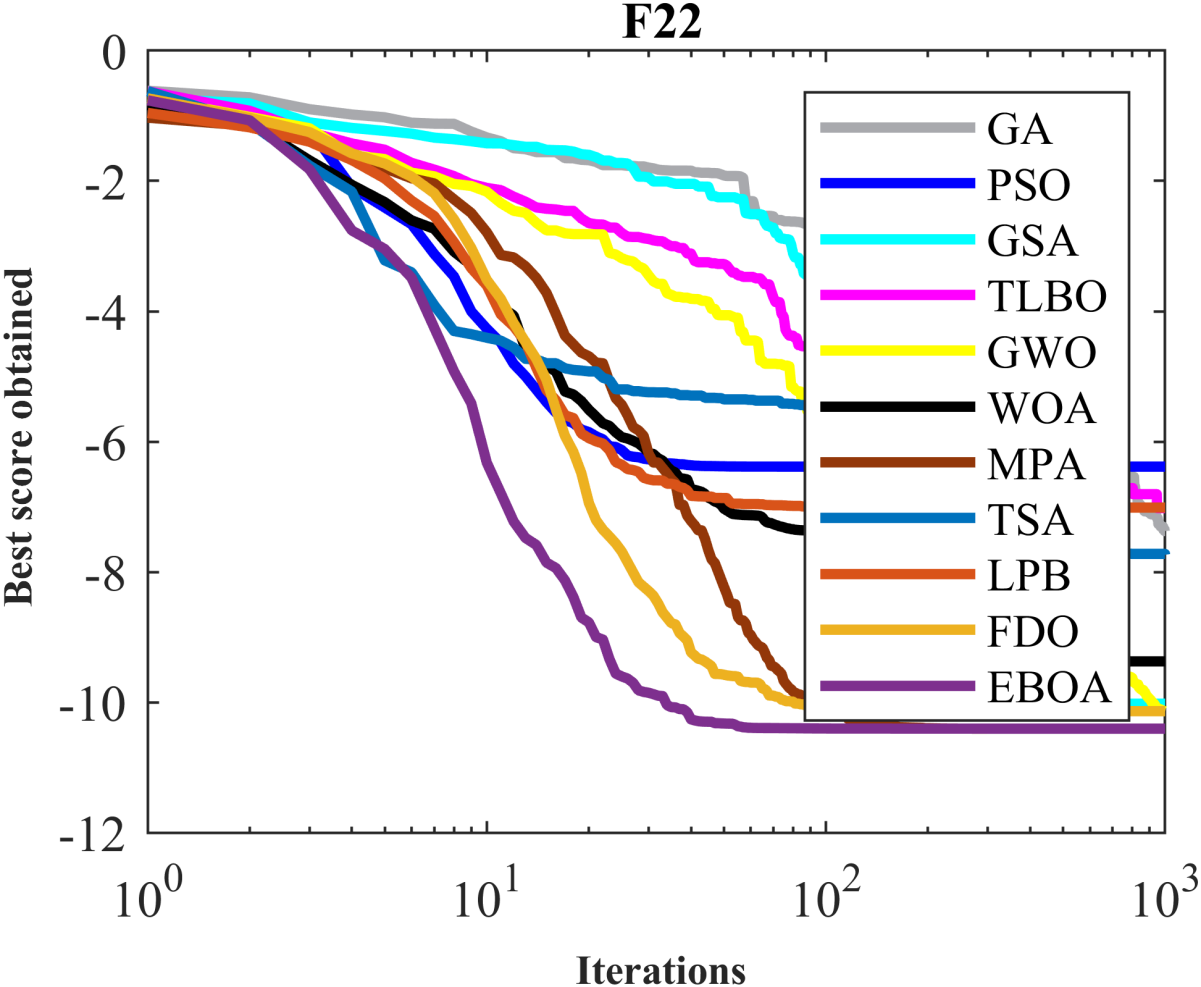
Convergence curves of EPOA and competitor algorithms on F22.

**Figure 11:**
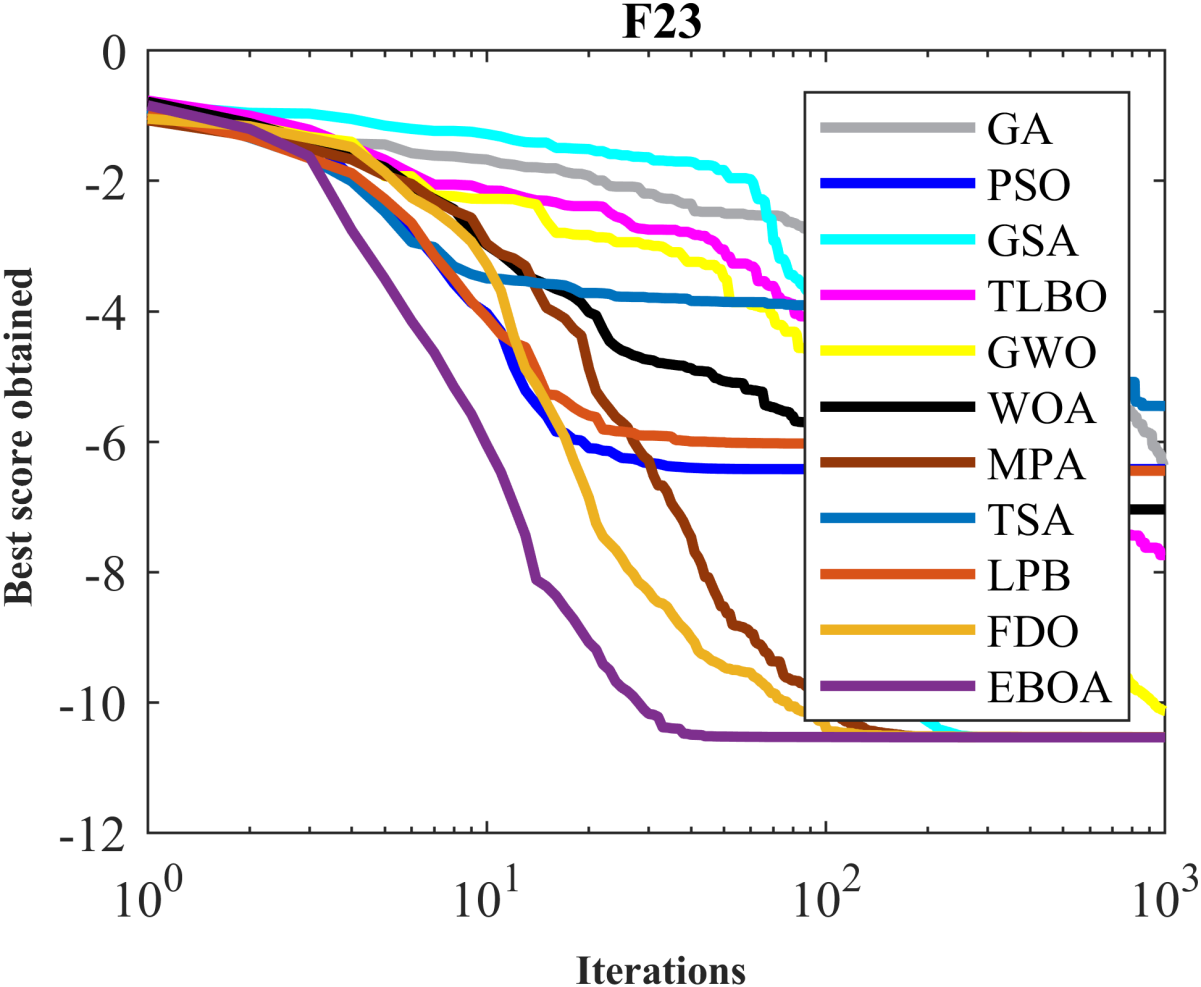
Convergence curves of EPOA and competitor algorithms on F23.

### Statistical analysis

Capability analysis of metaheuristic algorithms in terms of mean, best, std, median, and rank indices provides valuable information to compare their performance. However, a very small probability can be considered for the chance superiority of one method over another. In this study, the Wilcoxon rank sum test (Wilcoxon, 1992) and non-parametric *t*-test (Kim, 2015) are used to determine whether the superiority of the EBOA over any of the competing metaheuristic algorithms was statistically significant. The results of applying Wilcoxon rank sum test and non-parametric *t*-test on EBOA performance and competitor metaheuristic algorithms are released in Tables 9 and 10, respectively. In cases where the *p*-value is less than 0.05, it can be concluded that there is a significant difference between the two compared groups. What is clear from the results of the Wilcoxon rank sum test and non-parametric *t*-test is that the EBOA has a significant superiority in terms of statistical analysis over all ten competing algorithms in all objective function groups.

### Sensitivity analysis

The proposed EBOA approach is a population-based metaheuristic algorithm that addresses optimization problems in a repetitive-based process. Thus, the two parameters of EBOA, population number (*N*) and the maximum number of iterations of the algorithm (*T*), affect EBOA performance. This subsection is dedicated to the sensitivity analysis of EBOA to changes in *N* and *T* parameters.

**Figure 12:**
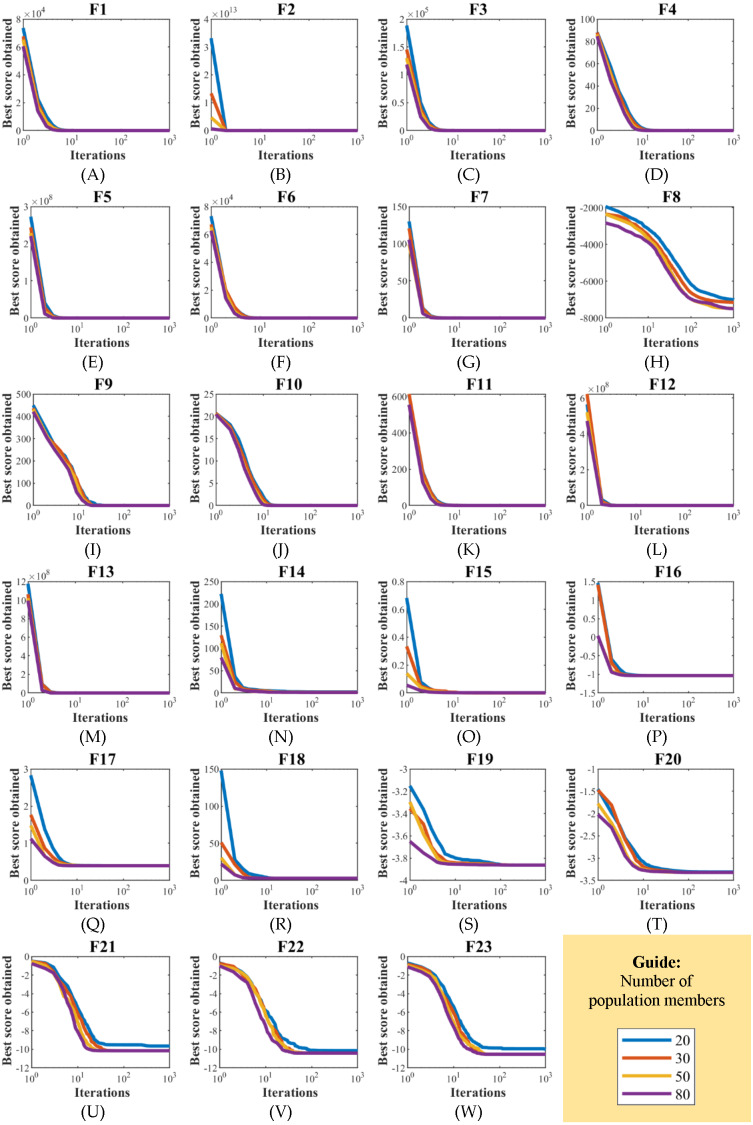
(A–T) EBOA convergence curves in the study of sensitivity analysis to population size N changes.

EBOA sensitivity analysis to parameter *N* has been studied by applying it to the handling of functions F1 to F23 for different values of parameter *N* equal to 20, 30, 50, and 80. The results of EBOA sensitivity analysis to parameter *N* are released in Table 11. The effect of the parameter *N* changes on EBOA convergence curves and how to achieve the solution is shown in Fig. 12. The simulation results reveal the fact that increasing the EBOA population size increases the search power of this algorithm, as it can be seen that by increasing the values of parameter *N*, the proposed EBOA achieves better solutions and as a result, the values of all objective functions decrease.

**Figure 13:**
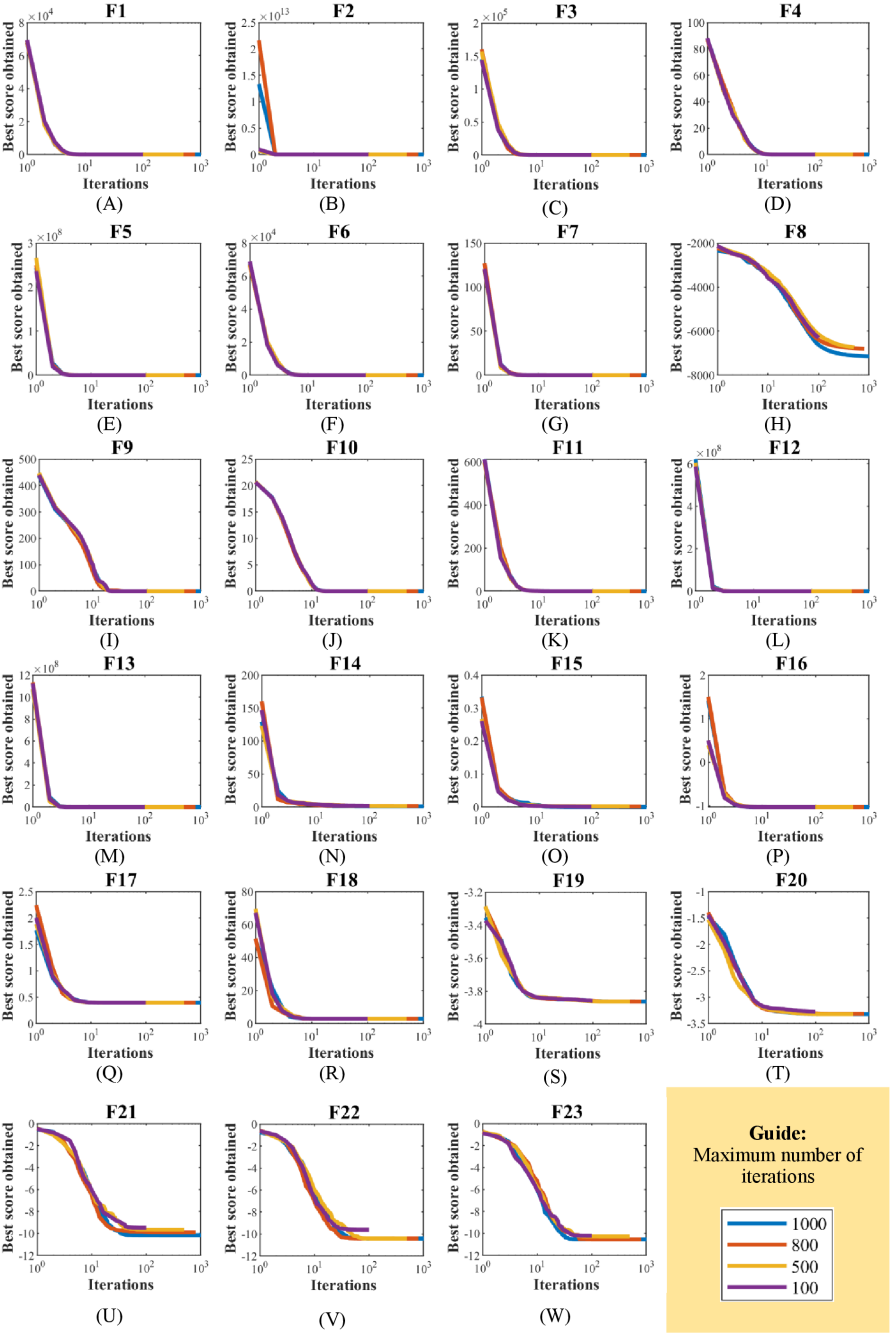
(A–W) EBOA convergence curves in the study of sensitivity analysis to maximum number of iterations T changes.

EBOA sensitivity analysis to the parameter *T* has been tested by implementing it on the handling of F1 to F23 functions for the parameter *T* equal to 100, 500, 800, and 1,000. The outputs of the EBOA sensitivity analysis for the *T* parameter value are shown in Table 12. In addition, the EBOA convergence curves, which show how to achieve the optimal solution under changes in the *T* parameter, are shown in Fig. 13. What can be understood from the simulation results is that increasing the number of iterations gives the EBOA more opportunity to be able to identify the main optimal area more accurately and to converge more towards the global optimal, which this reduced the values of the objective functions.

### Evaluation of CEC 2019 suite objective functions

The implementation of EBOA on the functions F1 to F23 indicated the high ability of EBOA in optimization applications. In this subsection, the performance of the EBOA is evaluated in addressing the CEC 2019 objective functions, which consist of ten functions of CEC01 to CEC10. The optimization results of CEC 2019 functions using EBOA and competitor algorithms are presented in Table 13. EBOA is the first best optimizer in addressing the functions cec02, cec03, cec07, cec08, cec09, and cec10. The results of the Wilcoxon rank-sum test and *t*-test are reported in Table 14. In cases where the *p*-value in this table is less than 0.05, the proposed EBOA approach has a statistically significant superiority over the corresponding algorithm. Analysis of the simulation results shows that the proposed EBOA approach has a superior performance over competitor algorithms in handling most cases of CEC 2019 test functions.

## Discussion

Exploitation and exploration are very influential on the performance of metaheuristic algorithms in finding optimal solutions to problems. Exploitation is the notion of local search capability around existing solutions that enables the algorithm to converge to better solutions that may be located in situations close to existing solutions. The impact of exploitation on the ability of metaheuristic algorithms is especially evident in dealing with problems that have only one main peak. The results of optimizing the functions F1 to F7 (with only the main peak) show that the EBOA has the high exploitation ability in local search and convergence to the global optimal solution. The high exploitation of the EBOA is especially evident in the handling of the functions F1, F3, and F6, which has converged to the global optimal.

Exploration is the concept of global search capability in all areas of the problem-solving space that enables the algorithm to identify the main optimal area containing the global optimal in the presence of local optimal areas. The effect of exploration on the ability of metaheuristic algorithms is especially evident in handling problems that have several non-optimal peaks in addition to the main peak. The results of optimizing the F8 to F13 functions (with several non-optimal peaks) show that the EBOA has acceptable exploration power in the global search and identification of the main optimal area. The high exploration capability of EBOA, especially in handling F9 and F11, has led to the accurate identification of the main optimal area and the success of the algorithm in achieving the global optimum.

**Table 9:** Results of applying Wilcoxon rank sum test on performances of EBOA and competitor metaheuristic algorithms.

Compared algorithm	Objective function type		
	Unimodal	High-dimensional multimodal	Fixed-dimensional multimodal
EBOA *vs.* GA	1.01E−24	4.02E−18	1.04E−22
EBOA *vs.* PSO	1.01E−24	2.42E−20	3.74E−34
EBOA *vs.* GSA	9.78E−25	1.89E−21	1.28E−32
EBOA *vs.* TLBO	9.3E−21	3.51E−12	4.35E−33
EBOA *vs.* GWO	6.49E−23	6.96E−08	1.46E−24
EBOA *vs.* WOA	1.07E−13	4.58E−11	0.018214
EBOA *vs.* TSA	1.78E−20	2.37E−12	0.044185
EBOA *vs.* MPA	1.01E−24	5.53E−06	1.44E−34
EBOA *vs.* LPB	1.35E−21	0.0002	7.37E−31
EBOA *vs.* FDO	6.98E−12	6.05E−07	6.24E−15

**Table 10:** Results of applying non-parametric t-test on performances of EBOA and competitor metaheuristic algorithms.

Compared algorithm	Objective function type		
	Unimodal	High-dimensional multimodal	Fixed-dimensional multimodal
EBOA *vs.* GA	0.018107	0.019062	0.034177
EBOA *vs.* PSO	6.78E−06	4.4E−06	1.45E−09
EBOA *vs.* GSA	1.25E−06	2.9E−06	0.015419
EBOA *vs.* TLBO	5.47E−06	3.01E−05	2.5E−13
EBOA *vs.* GWO	1.61E−06	4.42E−06	1.17E−11
EBOA *vs.* WOA	7.43E−06	0.00133	0.001565
EBOA *vs.* TSA	4.73E−06	0.031683	1.12E−10
EBOA *vs.* MPA	9.96E−06	5.93E−06	2.5E−07
EBOA *vs.* LPB	0.024004	0.075963	4.85E−11
EBOA *vs.* FDO	6.64E−08	0.001498	9.29E−13

**Table 11:** Results of EBOA sensitivity analysis to parameter N.

Objective functions	Number of population members			
	20	30	50	80
F_1_	0	0	0	0
F_2_	2.4E−210	1.3E−261	1.2E−291	0
F_3_	0	0	0	0
F_4_	4.2E−214	5.3E−260	1.1E−284	3.4E−304
F_5_	26.39773	25.91771	25.51165	24.81
F_6_	0	0	0	0
F_7_	6.73E−05	4.77E−05	3.04E−05	1.99E−05
F_8_	−7006.16	−7149.45	−7477.15	−7491.36
F_9_	0	0	0	0
F_10_	2.49E−15	1.24E−15	8.88E−16	8.88E−16
F_11_	0	0	0	0
F_12_	4.4E−07	2.71E−07	1.77E−07	1.1E−07
F_13_	0.001125	3.88E−06	2.87E−06	1.6E−06
F_14_	2.432658	0.998	0.998	0.998
F_15_	0.000379	0.000308	0.000307	0.000307
F_16_	−1.03163	−1.03163	−1.03163	−1.03163
F_17_	0.397887	0.397887	0.397887	0.397887
F_18_	3	3	3	3
F_19_	−3.86278	−3.86278	−3.86278	−3.86278
F_20_	−3.31004	−3.322	−3.322	−3.322
F_21_	−9.64339	−10.1532	−10.1532	−10.1532
F_22_	−10.1371	−10.4029	−10.4029	−10.4029
F_23_	−9.98805	−10.5364	−10.5364	−10.5364

**Table 12:** Results of EBOA sensitivity analysis to parameter T.

Objective functions	Maximum number of iterations			
	100	500	800	1,000
F_1_	3.52E−47	4.5E−263	0	0
F_2_	1.09E−22	1.4E−125	8.9E−209	1.3E−261
F_3_	1.08E−41	3.3E−231	0	0
F_4_	9.21E−24	2.6E−127	3.9E−206	5.3E−260
F_5_	28.41803	27.17727	26.53139	25.91771
F_6_	0	0	0	0
F_7_	0.000665	9.19E−05	5.67E−05	4.77E−05
F_8_	−6297.42	−6741.15	−6801.7	−7149.45
F_9_	0	0	0	0
F_10_	2.15E−15	2.14E−15	2.13E−15	1.24E−15
F_11_	0	0	0	0
F_12_	0.001396	2.55E−06	1.02E−06	2.71E−07
F_13_	0.068494	2.27E−05	2.04E−05	3.88E−06
F_14_	0.998	0.998	0.998	0.998
F_15_	0.000924	0.002341	0.001376	0.000308
F_16_	−1.03163	−1.03163	−1.03163	−1.03163
F_17_	0.397889	0.397887	0.397887	0.397887
F_18_	3	3	3	3
F_19_	−3.8582	−3.86278	−3.86278	−3.86278
F_20_	−3.27088	−3.29773	−3.30403	−3.322
F_21_	−9.47147	−9.53358	−9.64336	−10.1532
F_22_	−9.60492	−10.4029	−10.4029	−10.4029
F_23_	−10.2011	−10.4668	−10.5364	−10.5364

In addition to having high capabilities in exploration and exploitation, the conditions that predispose metaheuristic algorithms to success in achieving solutions are the proper balance between these two indicators. Objective functions F14 to F23 have fewer non-optimal peaks than functions F8 to F13, and are good criteria for analyzing the ability of optimization algorithms to have the proper balance between exploration and exploitation. The results of optimization of F14 to F23 functions indicate that EBOA has a high potential for balancing exploration and exploitation to identify the main optimal region and converge towards the global optimal. An overall analysis of the results of optimizing the F1 to F23 objective functions frees the inference that the proposed EBOA approach has a high potential for exploration and exploitation as well as a balance between the two capabilities.

## Conclusions

Metaheuristic algorithms are one of the most widely used and effective stochastic methods for solving optimization problems. In this study, a new human-based algorithm called the Election Based Optimization Algorithm (EBOA) was proposed. The fundamental inspiration of the EBOA is the voting and election process in which people vote for their preferred candidate to elect the leader of the population. The EBOA steps in two phases of (i) exploration, including election holding and (ii) exploitation, including raising public awareness for better decision-making are mathematically modeled. The efficiency of EBOA in providing solutions to optimization problems was tested on thirty-three standard benchmark functions of a variety of unimodal, high-dimensional multimodal, fixed-dimensional multimodal, CEC 2019 types. The optimization results of unimodal functions indicated the high exploitation ability of EBOA in local search. The optimization results of high-dimensional multimodal functions showed the EBOA exploration capability in the global search of problem-solving space. In addition, the results obtained from the optimization of fixed-dimensional multimodal functions concluded that EBOA, by creating the proper balance between exploration and exploitation, has an effective efficiency in providing solutions to this type of problems. The implementation of EBOA on the complex CEC2019 suite test functions indicated the effectiveness of the proposed approach in dealing with complex optimization problems. The quality of the results delivered by the EBOA is compared against the performance of ten state-of-the-art metaheuristic algorithms. Comparing the simulation results, it can be found that EBOA has provided better optimization results and is much more competitive than the ten metaheuristic algorithms. The findings of simulation, statistical analysis, and sensitivity analysis indicate the high capability and efficiency of the EBOA in dealing with optimization issues.

**Table 13:** Optimization results of EBOA and competitor metaheuristics on CEC 2019 suite test.

		**EBOA**	**FDO**	**LPB**	**MPA**	**TSA**	**WOA**	**GWO**	**TLBO**	**GSA**	**PSO**	**GA**
cec01	Mean	31397.63	15232289	2.89E+10	31395.7	1.65E+08	2.12E+10	79199216	4.33E+08	3.24E+12	4.6E+08	5.52E+10
	Best	31395.7	187157.2	1.57E+09	31395.7	39799.58	1773823	45782.62	101859.5	6.80E+11	4374858	6.23E+09
	std	5.282375	22511868	2.09E+10	0.000292	4.16E+08	2.76E+10	1.1E+08	7.95E+08	2.05E+12	1.36E+09	4.6E+10
	Median	31395.79	4646710	2.52E+10	31395.7	2266337	8.34E+09	35900762	1.01E+08	2.94E+12	69939138	4.1E+10
	Rank	2	3	9	1	5	8	4	6	11	7	10
cec02	mean	17.34286	17.34286	27.70728	17.34286	18.34769	17.34508	17.34332	17.37083	14774.91	17.34286	53.56418
	Best	17.34286	17.34286	17.34892	17.34286	17.34803	17.34302	17.34311	17.35692	7679.092	17.34286	19.23928
	std	0	2.61E−09	13.66182	7.89E−12	0.808127	0.001867	0.000124	0.008292	4550.232	3.05E−15	31.00016
	Median	17.34286	17.34286	21.74086	17.34286	18.17572	17.34464	17.3433	17.36932	14635.08	17.34286	41.51365
	Rank	1	4	9	3	8	6	5	7	11	2	10
cec03	mean	12.7024	12.7024	12.7024	12.7024	12.70296	12.7024	12.7025	12.70241	12.7024	12.7024	12.70241
	Best	12.7024	12.7024	12.7024	12.7024	12.7024	12.7024	12.7024	12.70241	12.7024	12.7024	12.7024
	std	3.65E−15	1.50E−11	5.55E−08	3.65E−15	0.001339	5.24E−07	0.000415	9.08E−06	3.65E−15	2.08E−15	1.85E−06
	Median	12.7024	12.7024	12.7024	12.7024	12.70247	12.7024	12.7024	12.70241	12.7024	12.7024	12.7024
	Rank	1	3	4	1	9	5	8	7	1	2	6
cec04	mean	29.40091	25.8849	79.20654	8.011005	4244.962	253.3125	264.7791	230.9865	6.51698	67.58687	134.1774
	Best	12.93446	7.960353	20.97703	0.00017	74.23499	110.3315	13.37336	161.5526	2.984877	10.94454	38.25033
	std	14.08728	8.968979	47.08096	4.384481	2404.606	154.3524	581.9962	52.66575	2.247511	116.2151	59.15384
	Median	26.36631	25.30171	70.53092	8.470501	4468.768	215.8693	57.17001	225.0158	6.964711	29.8487	135.3556
	Rank	4	3	6	2	11	9	10	8	1	5	7
cec05	mean	1.160508	1.111998	1.226455	1.050818	2.999024	1.576271	1.266711	1.857917	1.00948	1.193098	1.602569
	Best	1.051728	1.040138	1.048986	1.014772	1.871421	1.201734	1.058202	1.658814	1	1.063978	1.288733
	std	0.065115	0.038701	0.133284	0.032407	1.072619	0.251901	0.208595	0.138394	0.010012	0.208163	0.214074
	Median	1.137767	1.121755	1.193437	1.044283	2.632057	1.576226	1.189335	1.8488	1.007396	1.145125	1.552622
	Rank	4	3	6	2	11	8	7	10	1	5	9
cec06	mean	2.106428	8.140997	5.623278	1.818019	10.42986	8.978288	10.42138	10.59729	1.000105	3.993241	8.647723
	Best	1.15137	5.874072	4.320987	1.114883	9.163213	7.276666	9.009652	9.853189	1.000073	1.228136	5.785451
	std	0.660714	0.661779	0.807592	0.744312	0.692189	1.124288	0.783399	0.434776	1.97E−05	1.907946	1.341339
	Median	1.929794	8.217592	5.516918	1.404095	10.3353	8.741017	10.26319	10.6044	1.000105	4.0335	9.229273
	Rank	3	6	5	2	10	8	9	11	1	4	7
cec07	mean	112.4807	143.7672	237.0939	174.3536	617.3397	616.2879	418.7292	623.5747	187.8737	165.1417	124.3327
	Best	14.75369	90.65738	61.10223	110.9085	246.6122	148.1279	79.03335	304.7375	82.15282	10.37837	12.09356
	std	79.22918	32.18216	140.8724	45.33848	259.8789	349.1442	300.5297	178.8176	90.5322	132.6008	92.31307
	Median	112.4599	144.3338	215.906	152.9829	597.9233	632.1518	334.0758	603.1046	180.2679	148.4302	120.9174
	Rank	1	3	7	5	10	9	8	11	6	4	2
cec08	mean	2.546949	4.300242	5.419414	3.869595	6.139661	5.839999	4.761854	5.360152	5.372261	5.021396	5.04118
	Best	1.289734	3.083263	3.559119	2.708939	4.895428	4.841095	2.925632	4.301028	4.363495	3.633744	4.04053
	std	0.727627	0.610021	0.598187	0.616791	0.472164	0.523359	0.942676	0.737183	0.499415	0.644997	0.466454
	Median	2.703282	4.336097	5.419597	4.034933	6.234195	5.892174	4.984088	5.222119	5.35066	5.112039	5.042585
	Rank	1	3	9	2	11	10	4	7	8	5	6
cec09	mean	2.343608	2.367272	3.13243	2.359083	440.234	4.575637	4.416888	19.60514	3.14272	2.549543	3.668416
	Best	2.33839	2.346984	2.720081	2.341292	2.896331	3.582951	3.596261	4.652537	2.576973	2.395394	2.819678
	std	0.005293	0.015551	0.288009	0.022234	591.7603	0.891569	0.651956	61.46722	0.494874	0.131925	0.509679
	Median	2.341382	2.362613	3.144558	2.35069	279.8453	4.582947	4.431976	5.863226	3.011512	2.514102	3.538552
	Rank	1	3	5	2	11	9	8	10	6	4	7
cec10	mean	5.313416	20.00304	20.03576	17.11663	20.4158	20.16707	20.43476	19.50824	18.64584	20.00118	19.3639
	Best	8.88E−16	19.91337	20.01024	0.000229	20.26639	20.04477	20.29121	9.348959	3.25E−09	19.99751	7.534284
	std	8.723522	0.041617	0.019828	7.057655	0.069939	0.102794	0.082589	2.869468	4.639721	0.006276	3.0762
	Median	1.15E−14	19.99981	20.03535	20	20.40561	20.14991	20.44391	20.38098	19.99088	19.99974	20.24951
	Rank	1	7	8	2	10	9	11	5	3	6	4
Sum rank		19	38	68	22	96	81	74	82	49	44	68
Mean rank		1.9	3.8	6.8	2.2	9.6	8.1	7.4	8.2	4.9	4.4	6.8
Total rank		1	3	5	2	10	8	7	9	5	4	6

**Table 14:** Results of applying the Wilcoxon rank sum test and non-parametric *t*-test on CEC 2019 test functions.

Compared algorithm	Test type	
	Wilcoxon rank sum test	*t*-test
EBOA *vs.* GA	1.21E−23	0.000446
EBOA *vs.* PSO	1.4E−12	0.14247
EBOA *vs.* GSA	1.01E−08	0.000112
EBOA *vs.* TLBO	9.19E−34	0.028878
EBOA *vs.* GWO	5.17E−26	0.007451
EBOA *vs.* WOA	2.66E−34	0.005366
EBOA *vs.* TSA	1.44E−34	0.09192
EBOA *vs.* MPA	0.244915	0.048768
EBOA *vs.* LPB	3.89E−27	0.000215
EBOA *vs.* FDO	4.12E−16	0.010523

The proposed EBOA approach enables several future directions, the most specific of which are the development of the EBOA binary version for discrete space applications, and the design of the EBOA multi-objective version to handle multi-objective optimization problems. The EBOA is applied to solve optimization problems in various sciences as well as real-world applications are other suggestions for future directions.

The proposed EBOA approach is a stochastic-based solving method. So, the main limitation of EBOA, similar to all stochastic-based approaches, is there is no guarantee that EBOA will achieve the optimal global solution. In addition, EBOA may fail to address some optimization applications because, according to the NFL theorem, there is no presumption that a metaheuristic algorithm is successful or not. Another limitation of EBOA is that it is always possible to develop newer algorithms that perform better than existing algorithms and EBOA. However, the optimization results show that the EBOA has provided solutions that are very close to the global optimal and, in some cases, precisely the global optimal. This EBOA capability is particularly evident in optimizing the F1, F3, F6, F9, F11, and F18 because it has made available the optimal global solution.

## Supplemental Information

10.7717/peerj-cs.976/supp-1 Supplemental Information 1 Matlab code of proposed algorithm EBOA A proposed algorithm that implements EBOA steps on optimization of objective functions and publishes the optimal solution in the output.

10.7717/peerj-cs.976/supp-2 Supplemental Information 2 File of code for objective functions Determines the information of the objective function and provides it to the main program.

10.7717/peerj-cs.976/supp-3 Supplemental Information 3 Input of the EBOA optimizer The main implementation code that call the information of the objective functions (including: number of variables, upper and lower band of variables, and mathematical formula of the objective function). This code then puts the objective function information as the input of the EBOA optimizer to determine its optimal solution.

## Additional Information and Declarations

### Data Availability

The following information was supplied regarding data availability: All our code for the new EBOA algorithm in MATLAB is available in the Supplemental Files.
